# Underlying Mechanism of Wild *Radix pseudostellariae* in Tolerance to Disease Under the Natural Forest Cover

**DOI:** 10.3389/fmicb.2020.01142

**Published:** 2020-05-27

**Authors:** Hongmiao Wu, Jinshen Xia, Xianjin Qin, Huiming Wu, Shengkai Zhang, Yanlin Zhao, Christopher Rensing, Wenxiong Lin

**Affiliations:** ^1^Fujian Provincial Key Laboratory of Agroecological Processing and Safety Monitoring, College of Life Sciences, Fujian Agriculture and Forestry University, Fuzhou, China; ^2^Key Laboratory of Crop Ecology and Molecular Physiology, Fujian Agriculture and Forestry University, Fuzhou, China; ^3^Key Laboratory for Genetics, Breeding and Multiple Utilization of Crops, Ministry of Education, College of Crop Science, Fujian Agriculture and Forestry University, Fuzhou, China; ^4^Fujian Provincial Key Laboratory of Soil Environmental Health and Regulation, College of Resources and Environment, Fujian Agriculture and Forestry University, Fuzhou, China

**Keywords:** soil sickness, plant–microbe interactions, rhizospheric dialog, consecutive monoculture, allelopathic

## Abstract

Replanting disease caused by negative plant-soil feedback in continuous monoculture of *Radix pseudostellariae* is a critical factor restricting the development of this common and popular Chinese medicine, although wild *R. pseudostellariae* plants were shown to grow well without occurrence of disease in the same site for multiple years. Therefore, we aimed to identify the changes in microbial community composition in the rhizosphere soil of wild *R. pseudostellariae* thus providing a potential method for controlling soil-borne diseases. We analyzed differences in soil physicochemical properties, changes in soil microbial community structure, and root exudates of wild *R. pseudostellariae* under different biotopes. And then, simple sequence repeats amplification was used to isolate and collect significantly different formae speciales of *Fusarium oxysporum*. Finally, we analyzed the pathogenicity testing and influence of root exudates on the growth of *F. oxysporum*. We found that the different biotopes of *R. pseudostellariae* had significant effects on the soil microbial diversity. The soil fungal and bacterial abundances were significantly higher and the abundance of *F. oxysporum* was significantly lower under the rhizosphere environment of wild *R. pseudostellariae* than under consecutive monoculture. The relative abundances of most genera were *Penicillium*, *Aspergillus*, *Fusarium*, *Nitrobacter*, *Nitrospira*, *Streptomyces*, *Actinoplanes*, and *Pseudomonas*. Venn diagram and LEfSe analyses indicated numerously specific microbiome across all the samples, and the numbers of specific fungi were higher than the shared ones in the four biotopes. Eight types of phenolic acids were identified across all the rhizosphere soils. Mixed phenolic acids and most of the examined single phenolic acids had negative effects on the growth of isolated pathogenic *F. oxysporum* strains and promoted the growth of non-pathogenic strains. Similarly, correlation analysis suggested that most of the identified phenolic acids were positively associated with beneficial *Pseudomonas*, *Nitrobacter*, *Nitrospira*, *Streptomyces*, and *Bacillus*. This study suggested that wild *R. pseudostellariae* was able to resist or tolerate disease by increasing soil microbial diversity, and reducing the accumulation of soil-borne pathogens.

## Introduction

In recent years, the increase in world population and global climate change have increased the intensity of food production in agricultural systems. Intensive agriculture has great contributions on the increase of food availability in the past decades (Muller et al., 2017). However, this widespread intensification and consecutive monoculture of farming practices, combined with conversion of natural ecosystems to agriculture, are causing a major decline in soil quality and biodiversity globally (Muller et al., 2017). As a result, this agricultural expansion faces more stringent problems of replanting disease or soil sickness. Replanting disease leads to the serious soil-borne diseases and a decline in crop yield, these plant species include cucumber (Zhou and Wu, 2012), watermelon (Zhao et al., 2010), peanut (Li et al., 2014), and various Chinese medicinal plants (Wu et al., 2015, 2017a; Wei et al., 2018). In particular, approximately 70% of medicinal plant species with tuberous roots develop severe replant disease with continuous monoculture regimens (Wu H. et al., 2016). In China, the annual economic losses of replanting disease could reach more than billions of US dollars per year.

*Radix pseudostellariae*, an important traditional Chinese medicinal plant, is mainly produced in Fujian and Guizhou Provinces in southeast and southwest China, respectively. In recent years, Zhenshen 2 and Shitai 1 *R. pseudostellariae* have become the primary varieties after years of screening and genetic breeding of wild *R. pseudostellariae*, and these have since been planted in the main production regions of Fujian and Guizhou Provinces. However, continuous monoculture of the cultivated varieties has facilitated in the development of replant disease, which significantly decreased the quality and biomass of underground tubers (Wu et al., 2017a). Interestingly, our years of observation have shown that the wild uncultivated *R. pseudostellariae* was mainly distributed under the natural forest cover with a lower growth rate and yield and higher disease resistance and without disease in the same sites for multiple years. Meanwhile, we have planted the cultivated varieties and wild *R. pseudostellariae* lines in the same field in Fujian province. We found the wildtype could grow well but produce less biomass compared to the cultivated varieties (Supplementary Figure S1). However, the underlying mechanism of the wild *R. pseudostellariae* lines in relation to resistance or tolerance of disease remains unclear, especially in the case of the medicinal plants.

Various studies have indicated that community structure and the exhibited functions of the soil microbiome determine soil quality and ecosystem sustainability (Schloter et al., 2003; Yang et al., 2017; Wu H. et al., 2019). Meanwhile, previous studies have shown the distinct difference in soil microbiome between disease conducive soils and disease suppressive soils (Gómez Expósito et al., 2017; Carrión et al., 2018). A growing body of evidence suggested that replant disease is a process by which plants alter the biotic and abiotic qualities of soil, disrupt the balance of soil microbial community structure, and cause pathogen accumulation in the rhizosphere of plants (Mangan et al., 2010; Zhou and Wu, 2012; Wei et al., 2018; Wu H. et al., 2019). In our previous studies, we found that consecutive monoculture of *R. pseudostellariae* greatly altered the structure of the rhizosphere microbial community with increasing population size of pathogenic *Fusarium oxysporum*, *Talaromyces helicus* and *Kosakonia sacchari*, but with a decrease in the abundance of potentially beneficial *Penicillium*, *Pseudomonas* spp., *Burkholderia* spp., and *Bacillus pumilus* diversity in the rhizosphere soil (Wu H. et al., 2016; Chen et al., 2017).

Studies have shown that the microbial communities in the rhizosphere soil are likely to be dependent on the type and composition of root exudates secreted by plants (Haichar et al., 2008; Chaparro et al., 2013). Likewise, our previous studies have indicated that the root exudates of *R. pseudostellariae* were able to favor specific pathogens at the expense of beneficial microorganisms and significantly increased the relative abundance of pathogenic *F. oxysporum* (Wu et al., 2017a; Wu H. et al., 2019). The *F. oxysporum* fungus, one of the top 10 fungal pathogens in molecular plant pathology (Kamoun et al., 2015), was shown to be a ubiquitous and primary soil-borne pathogen in the occurrence of *R. pseudostellariae* replant disease (Chen et al., 2017; Qin et al., 2017; Wu H. et al., 2019), causing vascular wilt in a wide range of 100 different host plants. However, the overall effect of wild *R. pseudostellariae* plants on the rhizosphere microenvironment via induction of shifts in root exudates as well as the specific soil microbial community composition remains unclear.

To address this uncertainty, we investigated the changes in microbial community composition and key root exudates of wild *R. pseudostellariae* plants under different biotopes; we also isolated *F. oxysporum*. We then assessed *F. oxysporum* pathogenicity and the influence of root exudates on the growth of *F. oxysporum* to explain how wild *R. pseudostellariae* influences the rhizosphere processes using microbiota and root exudates interactions, thereby alleviating or tolerating disease.

## Materials and Methods

### Soil Sampling

Wild *R. pseudostellariae* is mainly distributed in natural forest on mountains, which are generally distributed on a large-scale in the Langya Mountain of Chuzhou City, Anhui Province, China (118°11′–20′ E, 32°14′–20′ N). The mean annual precipitation of the Langya Mountain is 1,050 mm, the mean annual temperature is 15.2°C, the frost-free period is 217 days, and the relative humidity is 75%. The vegetation is composed of subtropical evergreen deciduous forests and broad-leaf forests. The soil mainly consists of brown soil, yellow-brown soil, and limestone soil. Brown soil and limestone soil are widely distributed. We chose four elevation sites (110, 120, 154, and 178 m) along the similar slope gradients based on the main distribution community of wild *R. pseudostellariae* (Supplementary Figure S1D), and the samples sites were named chu2, chu4, chu3, and chu5 in August 2017. At each elevation position, three 50 × 50 cm plots were established to cover all the *R. pseudostellariae*. Rhizosphere soil samples were collected from five random locations within each plot and mixed together to form a composite sample. Rhizosphere soil samples of each elevation position were conducted in three replicates. In order to compare the rhizosphere microbial abundance between wild and cultured *R. pseudostellariae*, we also collected the rhizosphere soils of 1-year (FY) and the 2-year (SY) monoculture of *R. pseudostellariae* under field cultivation at Zherong, Fujian Province (119°89′ E, 27°25′ N), respectively. Rhizosphere soil adhering to the roots and rhizomes was brushed off and collected, and then the sampled soil of wild *R. pseudostellariae* was immediately sieved through a 2-mm nylon mesh to remove stones and plant residues. Next, one group of soil samples was stored at −80°C for DNA analysis, whereas another was kept at normal room temperature before air-drying for soil physicochemical property analysis.

### Soil Physicochemical and Biological Characteristics

We determined the soil NO_3_^–^-N (LOQ: 100 μg/kg, soil nitrate nitrogen detection kit, Suzhou Comin), NH_4_^+^-N (LOQ: 800 μg/kg, soil ammonium nitrogen detection kit, Suzhou Comin), cellulase activity (LOQ: 1 mg/d/g, Soil cellulase kit, Suzhou Comin), chitinase activity (LOQ: 15 μg/d/g, Soil chitinase kit, Suzhou Comin), and sucrase activity (LOQ: 0.5 mg/d/g, Soil sucrase kit, Suzhou Comin) using the Soil Kit, following the manufacturer’s protocols. Three independent replicate assays were extracted and measured for each treatment.

### Genomic DNA Extraction, PCR Amplification and Miseq Sequencing

Total DNA was extracted from 0.7 g subsamples of soil using a BioFast soil Genomic DNA Extraction kit (BioFlux, Hangzhou, China) based on the manufacturer’s protocol. The internal transcribed spacer (ITS1) and the variable regions 3 to 4 (V3–V4) were used to amplify the fungal-specific fragment and bacterial-specific fragment with the primers ITS1F/ITS2R (Supplementary Table S1) (Yao et al., 2017) and 341F/806R (Ikoyi et al., 2018), respectively. The PCR reactions were conducted using the Phusion High-Fidelity PCR Master Mix (New England Biolabs, Ipswich, United States) and the PCR products were purified using a Qiagen Gel Extraction Kit (Qiagen, Germany). The library was then sequenced on an Illumina HiSeq 2500 platform by Personal Biotechnology Co., Ltd. (Shanghai, China).

FLASH tool was used to merge paired-end reads (Magoc and Salzberg, 2011), then quality filtering and chimera removal was performed (Edgar et al., 2011; Bolger et al., 2014). The remaining sequences were used to perform OTU clustering and species annotation. The fungal and bacterial species annotation were determined via the Unite database (Koljalg et al., 2013) and Silva database (Quast et al., 2013), respectively. Alpha diversities were analyzed by Mothur version 1.31.2 (Schloss et al., 2009). The raw data have been deposited in the NCBI SRA database with the submission accession PRJNA622661.

### Quantitative PCR Analysis of Soil Fungal and Bacterial Abundance

Quantitative PCR assay was performed on the CFX96 Real-Time system (Bio-RAD, United States) to determine the abundances of total fungi (ITS1F/ITS4) (Gao et al., 2008; Okubo and Sugiyama, 2009), total bacteria (Eub338/Eub518) (Kielak et al., 2008; Weedon et al., 2012) and *Fusarium oxysporum* (ITS1F/AFP308R) (Lievens et al., 2005), respectively. The PCR primers conditions were listed in Supplementary Table S1. The standard curves were created using serial 10-fold dilutions of plasmids containing the target genes of total fungi, total bacteria and *F. oxysporum*. Four independent assays were performed for each treatment.

### Phenolic Acids Extraction and Determination

Soil phenolic acids were extracted as previously described (Wu H. et al., 2016). The Waters HPLC system equipped with a C18 column (Inertsil ODS-SP, 4.6 × 250 mm, 5 μm) was used to analyze phenolic acids. The mobile phase was a mixture of methanol and 2% acetic acid. Detection was performed at 280 nm and 30°C. Identification and quantification were confirmed by comparing retention times and areas with pure standards.

### Isolation and Validation of *F. oxysporum* and Pathogenicity Testing

Potato dextrose agar (PDA) containing 0.1 mg/mL streptomycin sulfate was used to isolate *F. oxysporum* from the rhizosphere soil of wild *R. pseudostellariae*. Total genomic DNA was extracted from all pure-culture isolates of *F. oxysporum* using the CTAB method. Genomic DNA was amplified using ITS1F to ITS4 primers (Supplementary Table S1), then subjected to sequencing and species determination. Then, simple sequence repeats (SSR) amplification was used to investigate genetic diversity of formae speciales of *F. oxysporum*. SSR detection was performed using FolSSR-3 primers (Mahfooz et al., 2012). PCR assays were conducted in a 20-μL volume mix containing 10 μL of Taq PCR Master Mix (2×), 1 μL of each primer, and 20 ng of purified DNA extracts. The PCR conditions were as follows: 94°C for 3 min; followed by 35 cycles of 94°C for 45 s, 50°C for 45 s, 72°C for 45 s; and a final elongation at 72°C for 10 min. The PCR amplicons were resolved by electrophoresis on a 3% agarose gel to identify the informative SSR loci across all isolates.

Based on the results of SSR, we selected the discrepant formae speciales of *F. oxysporum*, and added mycelia of *F. oxysporum* to the tissue culture of wild *R. pseudostellariae* in the M519 medium (supplemented with agar at 8 g/L, sucrose at 30 g/L, 6-BA at 0.3 mg/L). Then, the tissue cultures were placed in a growth chamber (day length 16 h, day/night temperature 26°C/18°C) for 15 days to observe the growth status and disease symptoms of *R. pseudostellariae*.

### Influence of Phenolic Acids on the Growth of *F. oxysporum*

The standardized phenolic acids used in this study to form a mixed solution (i.e., gallic acid:coumaric acid:protocatechuic acid:p-hydroxybenzoic acid:syringic acid:vanillin:ferulic acid:benzoic acid = 2.7:29.0:7.1:41.7:110.7:57.5:16.3:98.2) based on the soil measurements of phenolic acids in the wild *R. pseudostellariae* rhizosphere (chu2, chu4, chu3, and chu5). A series of concentration gradients of the mixed and single phenolic acids in the solution were set at 0, 10, 30, 60, 80, and 100 μmol/L. The culture medium contained 1/4 PDA and a series of concentration gradients of phenolic acids. Following preparation of the 1/4 PDA-phenolic acids plates, the fungal mycelia had been removed from actively growing colony margins and were inoculated in the center of each plate, then placed in an incubator at 30°C constant temperature for 7 days, with the final mycelium diameter recorded being taken at the end of the experiment. Each treatment was performed independently three times.

### Statistical Analysis

The R software package^1^ (version 3.6.1) was used to perform all of the statistical analyses, unless otherwise indicated. Faith’s phylogenetic metrics were used to calculate the alpha diversities of bacterial and fungal communities at the OTU level. Principal component analysis (PCA) and pair-group method with arithmetic mean (UPGMA) were used to analyze fungal and bacterial communities. The data were visualized using Circos^2^ (Version 0.69). To ensure the accuracy and reduce the complexity of calculation, genera with low abundances were eliminated when they constituted <0.005% of the average relative abundance across all of the samples. The co-occurrence networks were then generated using “igraph” packages (Csardi and Nepusz, 2006) and visualized by the “Gephi” interactive platform^3^ (Ver 0.9.2). Keystone species (nodes), which are defined as those that hold communicating nodes together, were identified by betweenness centrality values (Vick-Majors et al., 2014) and co-occurrence networks visualization analysis was performed using the Gephi platform. The correlations between microbial taxa and soil physicochemical characteristics were then evaluated using Spearman’s rank correlation test.

## Results

### Soil Physicochemical and Biological Characteristics

After assessing the rhizosphere soil variation of wild *R. pseudostellariae* at different elevations, we determined that different biotopes of *R. pseudostellariae* had statistically significant (*p* < 0.05) effects on the soil physicochemical characteristics (Table 1). The growth status and population quantity of *R. pseudostellariae* were showed in Supplementary Figure S2. The pH of chu5 and chu4 were 6.73 and 7.31, respectively, which are placed in the middle of all the samples. The NO_3_^–^-N and NH_4_^+^-N contents were the highest in the chu5, followed by those in the chu3. Chitinase content made up the majority in chu4 and chu5, while cellulose content had the highest value in chu3. Sucrase was significantly different (*p* < 0.05) among all four samples and could be arranged in decreasing order as chu4 > chu2 > chu3 > chu5.

**TABLE 1:** Soil chemical properties in the rhizosphere of *R. pseudostellariae*.

**Treatments**	**pH**	**NO_3_^–^-N (mg kg^–1^)**	**NH_4_^+^-N (mg kg^–1^)**	**Chitinase (mg *N*-acetyl-D-(+)-glucosamine g^–1^ soil d^–1^)**	**Cellulase (mg glucose g^–1^ soil d^–1^)**	**Sucrase (mg glucose g^–1^ soil d^–1^)**
chu2	7.49 ± 0.10^a^	34.81 ± 0.41^c^	25.58 ± 0.28^b^	11.44 ± 0.35^c^	6.29 ± 0.30^c^	52.46 ± 0.10^b^
chu4	7.31 ± 0.25^a^	30.31 ± 0.66^d^	24.55 ± 0.07^c^	18.40 ± 0.21^a^	6.62 ± 0.21^c^	53.05 ± 0.12^a^
chu3	5.25 ± 0.21^b^	40.34 ± 0.34^b^	25.20 ± 0.25^b^	8.74 ± 0.21^d^	10.87 ± 0.03^a^	51.98 ± 0.13^c^
chu5	6.73 ± 0.10^c^	52.97 ± 0.43^a^	35.43 ± 0.30^a^	14.46 ± 0.25^b^	7.76 ± 0.07^b^	51.42 ± 0.28^d^

*Row entries in each column with the different alphabet letters suffixed to them are significantly different (*p* < 0.05, *n* = 3).*

### Soil Fungal and Bacterial Abundance

**FIGURE 1:**
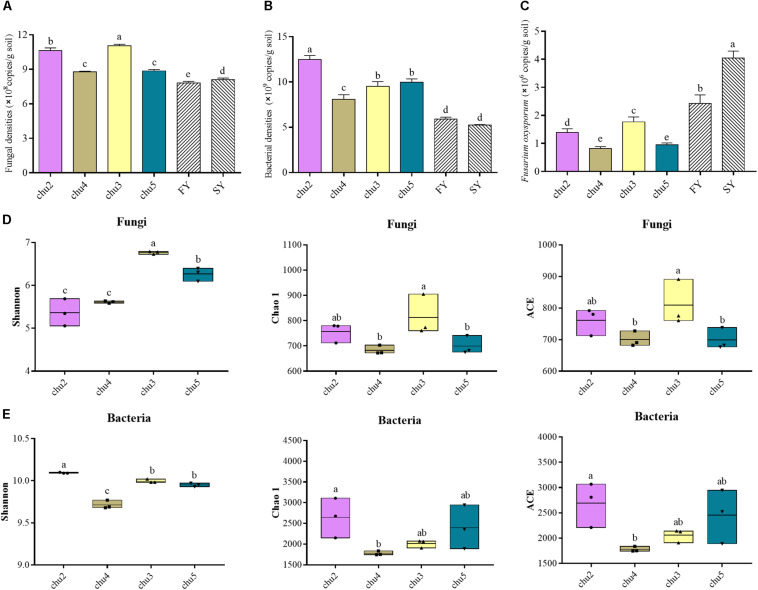
The abundances and alpha diversity indices of the microbial communities under different sampling sites. **(A–C)** represent the qRT-PCR results of fungal, bacterial and *F. oxysporum* abundance, respectively. **(D)** and **(E)** represent the high-throughput sequencing results of microbial community. FY and SY represent the 1- and 2-year monoculture of *R. pseudostellariae* under field cultivation, respectively. The different letters in each column represent significant differences (LSD-test, *p* < 0.05, *n* = 3).

The qRT-PCR analysis showed the abundances of soil fungi and bacteria were significantly (*p* < 0.05) higher under the wild *R. pseudostellariae* system than under consecutive monoculture (Figure 1). The abundances of *F. oxysporum* showed the opposite trend. In addition, chu3 and chu2 displayed a significantly higher fungal abundance across all the samples. These bacterial abundances were ranked in the following order: chu2, chu5, chu3, and chu4.

The sequencing of ITS1 and V3–V4 amplicons yielded a total of 393,257 and 418,625 effective tags, respectively. Moreover, the sequences of all samples were clustered to 7,642 fungal OTUs and 24,235 bacterial OTUs with a 97% identity threshold, with the number of OTUs per sample varying within the ranges of 599–673 and 1,777–2,241, respectively (Supplementary Figure S3). The alpha diversity indices of fungi and bacteria were represented by the Shannon, Chao1, and ACE indices (Figure 1). The results showed the fungal Shannon, Chao 1, and ACE indices of chu3 were significantly higher (*p* < 0.05) than the others. chu5 and chu4 displayed similar fungal Chao1 and ACE indices. Among the bacterial communities, the Chao1 and ACE indices of chu5 and chu2 were slightly but insignificantly (*p* > 0.05) higher than other samples. However, the bacterial Shannon of chu2 was the highest among all samples. In sum, different biotopes of *R. pseudostellariae* had significant effects on soil microbial alpha diversity.

### Beta Diversity of the Soil Microbial Community

**FIGURE 2:**
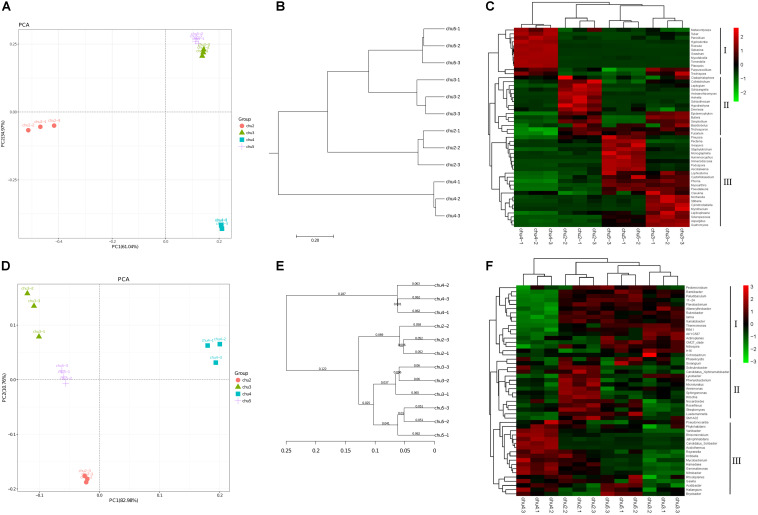
Beta diversity of the soil microbial community under different sampling sites. **(A,D)** Display Principal component analysis (PCA) of fungi and bacteria, respectively. **(B,E)** Display pair-group method with arithmetic mean (UPGMA) of fungi and bacteria, respectively. **(C,F)** Display hierarchically clustered heat map of the top 50 most abundant fungal and bacterial genera, respectively.

The PC1 and PC2 components explained 61.04 and 34.97% of the total fungal community variations, respectively (Figure 2). The fungal communities of chu5 and chu3 soils belonged to the same group. However, the chu2 and chu4 soils were located in the two groups, respectively, which were distinct from the other samples. Regarding analysis of the bacterial communities, the PC1 and PC2 components of PCA explained 82.98 and 10.76% of the total bacterial community variations, respectively. The result showed bacterial communities of chu5 and chu3 formed a group, the chu2 and chu4 were occupied by the two other groups.

### Shifts in Soil Microbial Community Structure

The fungal and bacterial taxon numbers at the phylum level were similar in different biotopes, and higher bacterial and lower fungal taxon numbers were observed in chu5 at the genus level (Supplementary Table S2). The predominant phyla of fungal community were Ascomycota, Basidiomycota, and Zygomycota, which accounted for more than 85% of the sequences, and *Basidiomycota* (71.69%) was the most abundant phylum in chu4. There were 29 bacterial phyla among the all samples (Supplementary Figure S4). Among the fungal orders, *Hypocreales* and *Eurotiales* showed the highest abundance in site chu3 compared to other samples. In the chu5 sample, the *Helotiales* made up the majority, followed by *Sordariales*, *Xylariales*, *Hypocreales*, and other orders (Supplementary Figure S5). Interestingly, there were less differences at the predominant bacterial order level among samples (Supplementary Figure S6). The hierarchical clustering heat map analysis showed that the relative abundances of significantly modified genera could be classified into three groups (Figure 2).

The relative abundances of most genera were *Penicillium*, *Aspergillus*, *Fusarium*, *Nitrobacter*, *Nitrospira*, *Streptomyces*, *Actinoplanes*, and *Pseudomonas*. The *Penicillium* and *Fusarium* showed the similarly relative abundances across all the samples (except chu4), and the *Aspergillus* was the highest in the chu3. The results also showed the contents of *Nitrobacter*, *Nitrospira*, and *Actinoplanes* were very similar between sites chu5 and chu2. Interestingly, the *Streptomyces* and *Pseudomonas* showed higher relative abundance in chu5 and chu2. The *Pseudomonas* accounted for 0.135 and 0.125% of the overall community dissimilarity in chu5 and chu2, respectively (Supplementary Table S3).

### Venn Diagram Analysis

**FIGURE 3:**
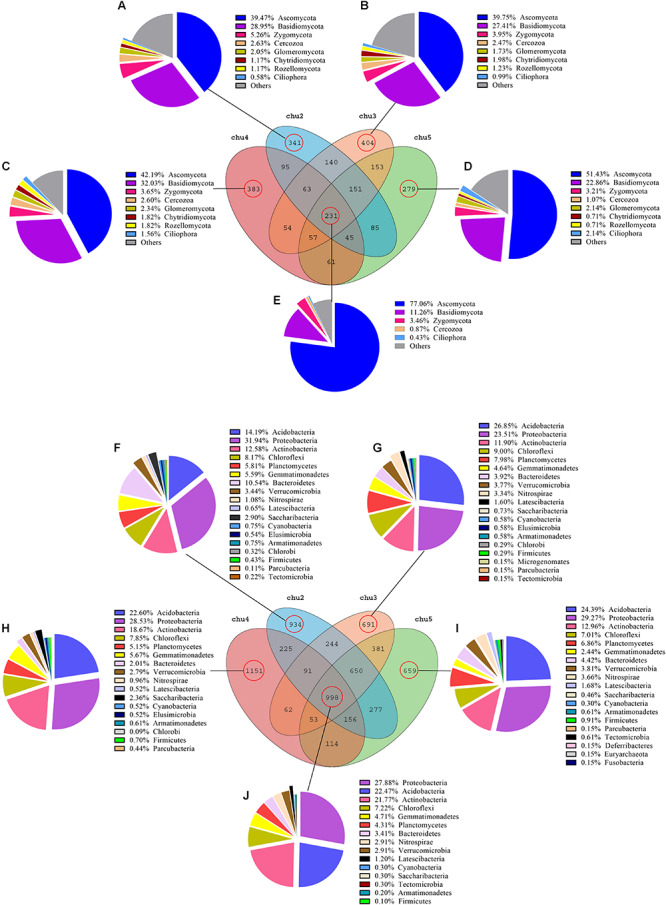
Venn diagram of exclusive and shared microorganism species-level taxa. **(A–E)**: fungal species; **(F–J)**: bacterial species.

Venn diagram analysis was conducted to detect exclusive and shared OTUs across all samples. The abundance of these 231 fungal OTUs shared in the all samples included Ascomycota (77.06%), Basidiomycota (11.26%), Zygomycota (3.46%), Cercozoa (0.87%), and Ciliophora (0.43%), respectively. The results showed that the number of fungal OTUs exclusively found in chu2, chu4, chu3, and chu5 were 341, 383, 404, and 279, respectively, and represented eight phyla (Figure 3). In addition, these genera could mainly be assigned to *Bullera*, *Basidiobolus*, *Tomentella*, and *Penicillium* (Supplementary Table S4). Furthermore, the results showed that the number of bacterial OTUs found in the all samples were up to 998 and represented 15 phyla that were mainly assigned to the Proteobacteria, Acidobacteria, and Actinobacteria (Figure 3). The number of bacterial OTUs exclusively found in chu2, chu4, chu3, and chu5 were 934, 1151, 691, and 659, respectively. Meanwhile, the shared and exclusive OTUs were mainly assigned to the genera *Haliangium*, *RB41*, *Roseiflexus*, *Acidothermus*, and *Gemmatimonas* (Supplementary Table S5).

### LEfSe and Co-occurrence Network Analysis

**FIGURE 4:**
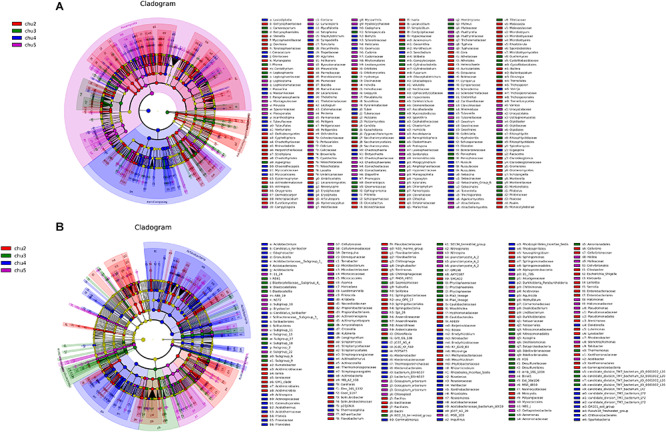
LEfSe results showing the phylogenetic structure of the microbiota from different samples. **(A)** Fungal communities. **(B)** Bacterial communities. There are five circular rings in the cladogram; each circular ring deposit all taxa within a taxonomic level; the circular ring from inside to outside represent phylum, class, order, family and genus, respectively. The node on the circular ring represents a taxon that is affiliated within the taxonomic level. The node size corresponds to the average relative abundance of the taxon. Taxa that had significantly higher relative abundance in a certain treatment within each soil type were color-coded within the cladogram.

LEfSe analysis was used to identify significantly different biomarkers (*p* < 0.05, LDA > 2.0) across all samples (Figure 4). The results showed numerous specific microbiomes across different elevation sites. The fungi with the greatest differences in numbers could be arranged in decreasing order as chu2 > chu4 > chu5 > chu3 and were affiliated with five phyla, which mainly belonged to the phyla Ascomycota and Basidiomycota, class *Saccharomycetes* and *Agaricomycetes*. The number of different bacterial biomarkers were affiliated with 15 phyla, which mainly comprised members of order *Rhizobiales*, *Burkholderiales*, *Micrococcales*, *Xanthomonadales*, *Myxococcales*, *Streptosporangiales*, and *Pseudomonadales*.

**FIGURE 5:**
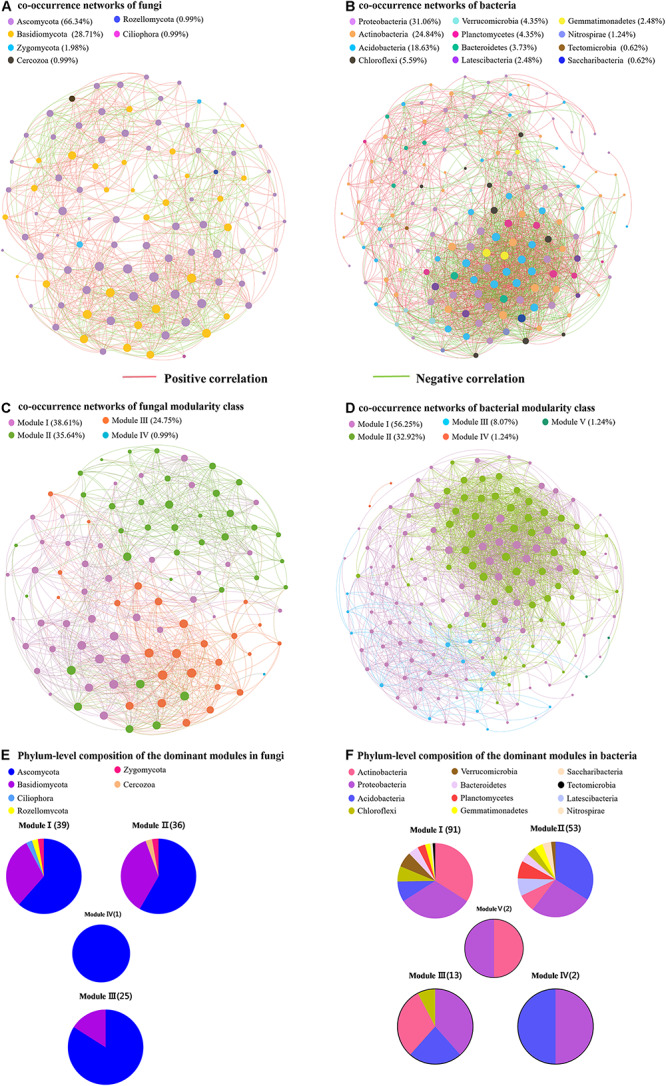
Network of co-occurring fungal and bacterial genera based on correlation analysis. **(A,B)** Represent the co-occurrence networks of fungi and bacteria, respectively. **(C,D)** Represent the co-occurrence networks of fungal and bacterial modularity class, respectively. **(E,F)** Represent phylum-level composition of the dominant modules in fungi and bacteria, respectively.

To compare the complexity of microbiome associations across all samples, four networks were constructed by combining all microbiomes originating from the four soil samples (Figure 5). The fungal network consisted of 101 nodes (genera) and 890 edges. These nodes were assigned to six fungal phyla and mainly affiliated to the Ascomycota, Basidiomycota, and Zygomycota (Figure 5A). When the distribution of nodes was modularized, all nodes were grouped into four major modules (Figure 5C). The nodes from modules I, II, and III mostly belonged to Ascomycota and Basidiomycota, with high abundances of the *Scleropezicula*, *Archaeorhizomyces*, *Bullera*, *Epidermophyton*, *Mycoarthris*, *Russula* genera (Figure 5E and Supplementary Figure S7). Based on the co-occurrence analysis for bacterial OTUs, 2,239 edges were captured from 161 nodes for the total soil samples. These nodes were assigned to 12 bacterial phyla (Figure 5B). Notably, as shown in Figure 5D, the entire bacterial network could be parsed into five major modules. Modules I and II accounted for 56.25 and 32.92%, respectively. The nodes from modules I and III mostly belonged to Actinobacteria, Proteobacteria, Acidobacteria, and Chloroflexi, respectively; nodes from module II were mostly Acidobacteria, Proteobacteria, Actinobacteria, Latescibacteria, and Planctomycetes (Figure 5F). The major module I and II included a high abundance of *Bryobacter*, *Candidatus*, *Gemmatimonas*, *Haliangium*, *Nitrobacter*, and *Variibacter* (Supplementary Figure S8). Meanwhile, the positive correlations of fungi and bacteria occupied 65.17 and 53.42%, respectively. Moreover, the respective average path length (APL) and clustering coefficient (CC) index of 2.335 and 0.601 in fungal networks, and 2.352 and 0.596 in bacterial networks strongly indicated that these observed networks have “small world” properties (Supplementary Table S6). The modularity (MD) indices of 1.354 and 6.135 (>0.4) in fungal and bacterial networks revealed that these networks have modular structures.

### Component Identification of Phenolic Acids in Rhizosphere Soil

**FIGURE 6:**
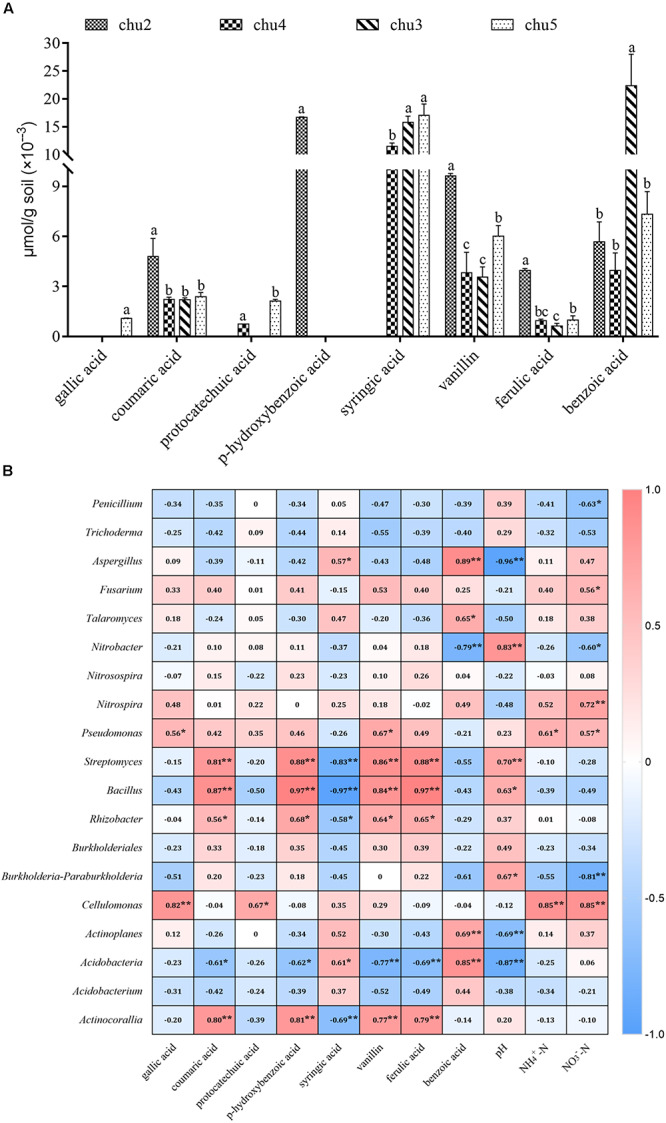
Changes in the levels of phenolic compounds in the rhizosphere soil **(A)** and the correlation analysis demonstrated the relationships between soil environmental factors and microbial communities **(B)**. Columns with different letters are statistically different (LSD test, *p* < 0.05, *n* = 3). **p < 0.01, *p < 0.05.

HPLC results showed eight types of phenolic acids were identified across all the soils, which included gallic acid, coumaric acid, protocatechuic acid, p-hydroxybenzoic acid, syringic acid, vanillin, ferulic acid, and benzoic acid (Figure 6A). The results showed that the predominant components were syringic acid, vanillin, benzoic acid, coumaric acid, and ferulic acid across all samples. The phenolic acids levels in the rhizosphere soil varied among biotopes.

### Correlation Analysis

The associations between microbial community structure and environment from different sites are shown in Supplementary Table S7 and Supplementary Figure S9. Among the variables, pH, and concentrations of chitinase and cellulose made the strongest contribution to the observed differences among the fungal and bacterial communities in all samples. In addition, NO_3_^–^-N and sucrose significantly affected the bacterial communities. RDA analyses demonstrated that soil NO_3_^–^-N and NH_4_^+^-N were positively correlated with a higher relative abundance of *Ascomycota*, *Proteobacteria*, *Tectomicrobia*, and *Bacteroidetes*, and negatively correlated with the lower abundance of *Basidiomycota*, *Actinobacteria*, and *Verrucomicrobia*. Strong associations were found between soil pH and the abundance of *Glomeromycota*, *Proteobacteria*, *Armatimonadetes*, and *Actinobacteria*.

Correlation analysis suggested that significant correlations based on the Spearman correlation coefficient were found between soil environmental factors and microbial taxa across all treatments (Figure 6B). The most frequently occurring phenolic acids were negatively correlated to potentially beneficial *Penicillium* and *Trichoderma* and positively associated with *Fusarium*, *Pseudomonas*, *Nitrobacter*, *Nitrospira*, *Streptomyces*, and *Bacillus*. Furthermore, soil pH was positively correlated with potentially beneficial *Penicillium* and *Trichoderma*, *Pseudomonas*, *Bacillus*, and *Rhizobacter*, and negatively correlated with pathogenic *Aspergillus*, *Fusarium*, and *Talaromyces*. The NO_3_^–^-N and NH_4_^+^-N contents were negatively correlated with *Penicillium*, *Nitrobacter*, *Trichoderma*, *Streptomyces*, and *Bacillus*, positively associated with *Fusarium*, *Talaromyces*, and *Aspergillus*.

### Validation of *F. oxysporum* Pathogenicity

**FIGURE 7:**
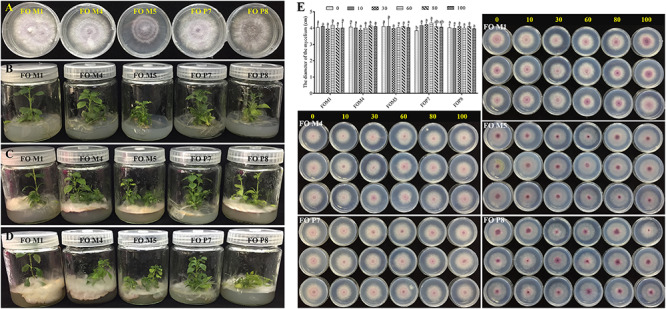
Validation of *F. oxysporum* pathogenicity **(A–D)** and the effects of phenolic compound mixture on the growth of five *Fusarium oxysporum* strains **(E)**. The yellow numbers represent the concentration of phenolic acids mixture (μmol/L). Columns with different letters are statistically different (LSD test, *p* < 0.05, *n* = 3).

The SSR markers were used to investigate genetic diversity among different-origin *F. oxysporum* (Supplementary Figure S10). The results showed that the cultivars and genetic resources of isolates collected from the wild were significantly different. In addition, there were obviously distinct distributions and number of bands among the five *F. oxysporum* strains. Since we inoculated the *F. oxysporum* strains (FOM4, FOM5, and FOP8) on the medium about 2–3 cm away from the roots of the tissue culture plantlets, different *F. oxysporum* strains initiated contact with roots about 3 days later, the leaves started to flatten and turn yellow after about 12 days, the basal part of the stems was covered with *F. oxysporum*, and the plants died 16 days later. The *F. oxysporum* strains (FOM4, FOM5, and FOP8) were highly pathogenic to the tissue culture plantlets of *R. pseudostellariae* (Figure 7). However, *F. oxysporum* strains (FOM1 and FOP7) were not pathogenic to the tissue culture plantlets.

### The Influence of Phenolic Acids on the Growth of *F. oxysporum*

We tested the influence of mixed and single phenolic acids on the physiological characteristics of five *F. oxysporum* strains (Figure 7E and Supplementary Figures S11–S18). The results showed that the mixture of phenolic acids significantly promoted the mycelial growth of non-pathogenic FOP7 when applied at a concentration from 10 to 60 μmol/L, and the positive effect decreased at high concentrations. A slight but insignificant promotions of pathogenic *F. oxysporum* strains (FOM4, FOM5, and FOP8) were observed at high concentrations, but these imparted inhibitory effects at low concentrations. Concurrently, it was found that each individual phenolic acid had different effects on the growth of the five *F. oxysporum* strains, with the predominant syringic acid significantly promoting the growth of the non-pathogenic FOM1 and FOP7. However, ferulic acid inhibited the growth of non-pathogenic *F. oxysporum* and had positive effects on some pathogenic strains (FOM4, FOM5, and FOP8). Syringic acid and benzoic acid promoted FOM5, and inhibited FOM4 and FOP8. Coumaric acid significantly exhibited a stimulatory effect on FOM5 and FOP8. Vanillin displayed positive effects on FOM1 and FOM4, but inhibited FOM5, FOP7, and FOP8 at a certain concentration. The rare p-hydroxybenzoic acid imparted significant inhibitory effects on pathogenic strains (FOM4 and FOP8). The rare protocatechuic acid and gallic acid inhibited FOP7 and FOP8, respectively.

## Discussion

### Enhanced Soil Microbial Biodiversity and Specific Microbiome Changes

In recent years, the land-use intensity has constantly increased at a global scale, resulting in the degradation of 25% of soils worldwide and threatening soil biodiversity (Stavi and Lal, 2015; Tsiafouli et al., 2015). This intensive agriculture system is characterized by high resource inputs and high losses, and thus has a depleted soil life (Bender et al., 2016). Generally, intensive agricultural practices are considered to lead to simpler soil food webs comprising of fewer functional groups and smaller-bodied organisms (Tsiafouli et al., 2015; Bender et al., 2016). Our previous results indicated that intraspecific intercropping of *R. pseudostellariae* increases fungal and bacterial community diversity, further reducing disease in the consecutive monoculture regimes (unpublished data). In this study, we found the fungal and bacterial abundances of wild *R. pseudostellariae* were significantly higher than the cultivated varieties in agricultural fields. This might be due to the fact that wild *R. pseudostellariae* is distributed in the natural ecosystems. Our data agrees with previous findings, which indicated natural ecosystems usually have a higher (sometimes much higher) level of soil biodiversity compared to agricultural land-use systems (Tuck et al., 2014; Tsiafouli et al., 2015). Meanwhile, Trap et al. (2016) found that the increase in bacterivorous microfauna contributed to enhanced plant nutrition. This indicated that these complicated plant–microbe interactions improved soil nutrient cycling.

We also found a number of specific microbiomes across different elevation levels, and the number of specific microbes was similar or higher than the shared ones in the four biotopes. Enhanced soil-specific microbiome changes can complement each other to increase overall ecosystem stability and sustainability (Bender et al., 2016). Furthermore, chitinase, cellulose, and pH were significantly correlated with soil fungal and bacterial community structure. Previous studies have shown that cellulase and chitinase were involved in the antagonistic activity of some biological control agents against soil-borne plant pathogens (Inbar and Chet, 1991; Budi et al., 2000; Qiuhong et al., 2006; Prasad et al., 2013). The findings of our study suggest that the wild *R. pseudostellariae* system increased both soil cellulose and chitinase levels compared to the cultivated varieties (Wu H. et al., 2019). The complex activity of soil enzymes may be involved in shaping and regulating microbial communities. Therefore, the rhizosphere of wild *R. pseudostellariae* has a high rate of internal regulatory processes, a rich soil life, and is characterized by low resource inputs and outputs.

### Wild *R. pseudostellariae* Preferentially Associated With the Rhizosphere Microbiome

In modern agriculture, cultural crops have been selected to maximize yield and confer additional benefits, particularly bringing in traits such as crop resistance to drought, pathogens, etc. (Mijatoviæ et al., 2013). In the selection process, less attention has been given to plant microbiomes, which have been shown to contribute to altered plant traits or disease suppression (Mendes et al., 2011; Bender et al., 2016), this is especially important because several modern plant cultivars have partially lost their ability to associate with beneficial soil biota (Sawers et al., 2008).

Among the communities of fungi and bacteria studied here, the predominant phyla included Ascomycota, Basidiomycota, Zygomycota, Proteobacteria, Acidobacteria, and Actinobacteria, and this relationship has been found in rhizosphere soils of different cultivation regions (Wu et al., 2016a; Wu H. et al., 2019; Wu L. et al., 2019). Network analysis also showed that most of the nodes belonged to these six phyla. These co-occurrence networks were explored to offer insights into microbial interactions. The associations in microbial networks may represent a niche or ecological interactions that are shared among microorganisms (Berry and Widder, 2014; Zhang et al., 2018). Our results showed the positive correlations of these microorganisms were significantly more frequent than the negative correlations. The positive correlations among microorganisms may be due to their ecological commensalism or mutualism (Berry and Widder, 2014). Moreover, the similar CC and path length were observed between the fungi and bacteria. The small path lengths are considered small-world networks and have been linked to the quick responses of an ecosystem to perturbations (Watts and Strogatz, 1998; Zhou et al., 2010; Zhang et al., 2018). Therefore, the fungi and bacteria of wild *R. pseudostellariae* rhizosphere microbial community may be equally sensitive to environmental changes.

The co-occurrence networks of microbial modules are densely linked network regions (Bissett et al., 2013). Similar to co-occurrence networks in other systems, the networks constructed here for the rhizosphere microbial communities of wild *R. pseudostellariae* exhibited modular characteristics. The MD values were higher in the bacterial networks than in the fungal networks. Several studies have interpreted modules as niches (Eiler et al., 2012; Wu et al., 2016b), and the higher MD values may therefore be linked to stronger niche differentiation in the rhizosphere bacteria than in the fungi. The modules in microbial networks were predominated by fungal (i.e., Ascomycota and Basidiomycota) or bacterial (i.e., Actinobacteria, Proteobacteria, and Acidobacteria) taxa. This is concordant with the result that the majority of the network links were derived from these microorganisms with other phyla. In modules of the network, *Gemmatimonas* can modulate C and N intakes, be involved in soil nutrient transformations, and contribute promoting plant growth and suppressing plant diseases (Carbonetto et al., 2014; Li et al., 2017). Beneficial *Nitrobacter* has been reported to contain genes encoding nitrite-oxidizing enzymes and provide increased N availability to plants (Shen et al., 2019). Overall, the microbial networks were comprised of highly connected fungi and bacteria and formed a “small world” topology in the rhizosphere of wild *R. pseudostellariae*. Furthermore, these strong ecological linkages manifesting as a cluster of commensalism or mutualism correlations, indicated that they may play critical roles in maintaining the structure and function of ecological communities. Hence, the complicated plant–microbiome interactions enriched in soil food webs, directly and indirectly affect the functions of the remaining soil biota.

### Wild *R. pseudostellariae* Supports Beneficial Microbes but Suppresses Host-Specific Pathogen Accumulation

Numerous studies have shown that the occurrence of replant disease, which is mediated by individual plant performance parameters, increases under conditions of abundant pathogen presence. Similarly, the relative abundance of pathogenic *Fusarium* significantly increased and beneficial *Pseudomonas* significantly decreased under consecutive monoculture of many crops such as *R. pseudostellariae*, *Panax notoginseng*, *Rehmannia glutinosa*, and *Panax quinquefolius*, these changes were considered to be the key factors limiting plant production under consecutive monoculture regimes (Jiao et al., 2015; Wu et al., 2015; Chen et al., 2017; Wei et al., 2018).

Root exudates initiate and modulate dialog between roots and soil microbiome, which has the propensity to shape the rhizosphere microbiome directly or indirectly and also influences the growth of plants (Li et al., 2014; Venturelli et al., 2015; Wu et al., 2017b). Extensive evidence suggests that the imbalanced microbial populations mediated by secreted phenolic acids are involved in allelopathy and cause replanting disease (Bais et al., 2006; Kulmatiski et al., 2008; Wu et al., 2015; Wu H. et al., 2016; Liu et al., 2017). We previously identified nine types of phenolic acids in the rhizosphere of cultivated varieties and in culture medium under sterile conditions (Wu H. et al., 2016), but eight types and lower phenolic acids content were detected in wild *R. pseudostellariae*. Previous results have also shown that phenolic acids with similar contents detected in soil significantly promoted the growth of soil-borne pathogenic *F. oxysporum* and inhibited the growth of beneficial *Pseudomonas* and *Bacillus pumilus* in the rhizosphere of *R. pseudostellariae* (Wu H. et al., 2016; Chen et al., 2017). Similar results were also observed in replanting disease in *Rehmannia glutinosa*, peanuts, and cucumber (Zhou and Wu, 2011; Li et al., 2014; Wu et al., 2015). However, the results of the present study showed that a mixture and also most of the single phenolic acids by themselves had negative effects on the growth of pathogenic *F. oxysporum* strains and promoted the growth of non-pathogenic strains. Meanwhile, we also found the abundance of *F. oxysporum* in the rhizosphere of wild *R. pseudostellariae* was far below the cultivated varieties in 2-year monocultured fields. Interestingly, correlation analysis suggested that the most often identified phenolic acids were positively associated with beneficial *Pseudomonas*, *Nitrobacter*, *Nitrospira*, *Streptomyces*, and *Bacillus*. This might be due to the lower types and concentrations of phenolic acids in rhizosphere soil of wild *R. pseudostellariae*. Previous studies have shown that the volatiles of the bacterial community had strong negative effects on the assembly of soil fungal colonizers (Li et al., 2020), and competitive interactions within soil beneficial bacterial communities could trigger the production of mVOCs (volatile organic compounds) that suppressed plant pathogenic fungi (De Boer et al., 2019). Our study implied that complex plant–microbe interactions may have suppressed host-specific pathogen accumulation in the rhizosphere of wild *R. pseudostellariae*.

Plant disease system are composed of pathogens and plant interactions in a specific environment, and the interactions of plants, pathogens, and environmental factors determine the occurrence and prevalence of diseases. In nature, the co-evolution between plants and pathogens was shown to result in multiple resistance of plants and pathogenic germination of physiological strains, which contribute to increase the genetic diversity of the host and pathogen population (Zhu, 2013). However, the pathogenic variations among pathogens involve targeted selection, which lead to the establishment of predominant pathogens with strong pathogenicity as the number of monoculture years increased, ultimately resulting in the occurrence of diseases in the continuous monoculture regimens (Zhu, 2013). For example, the disease variation rate of *Pyricularia grisea* is much faster than can be countered in resistant rice breeding (Zhu, 2013). A previous experiment corroborated the evolutionary hypothesis that increasing host diversity imposes disruptive selection on pathogen populations, suggesting that pathogen genotypes that are selectively favored by one host genotype may have lower fitness on other host genotypes in the mixture (Lambers et al., 2009; Marshall et al., 2009). Previous studies have also indicated that higher plants and microbial diversity contributed to reduce evolution in the associated *Phytophthora infestans* pathogen, leading to more stable pathogen population structure and weaker host selection in host–pathogen interactions, thus further reducing plant diseases (Yang et al., 2019). The existence of a large number of non-pathogenic physiological strains and their mutual recognition among hosts will inevitably affect the interaction between pathogenic strains and hosts, thus minimizing the occurrence of diseases (Zhu, 2013). Therefore, the presence of higher microbial biodiversity and less root exudates directionally selected, the less-pathogenic or non-pathogenic *F. oxysporum* strains in the rhizosphere of wild *R. pseudostellariae*, thus impeding the ability of the pathogenic fungus to evolve rapidly towards higher aggressiveness, which in turn impelled less malgenic symptoms in *R. pseudostellariae*.

## Conclusion

**FIGURE 8:**
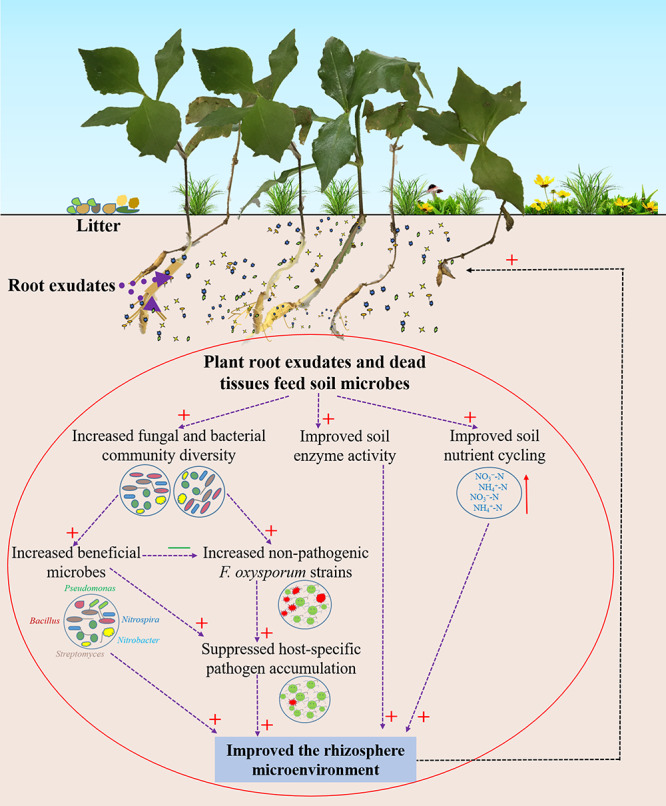
Schematic representation of rhizosphere responses to environmental conditions in the wild *R. pseudostellariae* rhizosphere under the natural forest. Red “+” represent a positive effect; green “–” represent a negative effect.

In summary, we provide evidence that wild *R. pseudostellariae* resists or tolerates disease by increasing soil microbial diversity and reducing the accumulation of soil-borne pathogens (Figure 8). Complex plant–microbe interactions promote the growth of beneficial microorganism and impede the coevolution of pathogenic *F. oxysporum* with the plant. Hence, the biodiversity or natural forest cropping systems may help in improving the performance and prevent replanting disease in *R. pseudostellariae*. Selection for plant microbiomes can also contribute to disease suppression.

## Data Availability Statement

The original contributions presented in the study are included in the article/Supplementary Material, further inquiries can be directed to the corresponding author/s.

## Author Contributions

WL and HoW conceived the study. HoW wrote the manuscript. HoW, JX, HuW, and SZ performed the experiments. HoW and YZ performed the statistical analyses. HoW and XQ were involved in soil sampling. CR and WL have revised the manuscript. All authors discussed the results and commented on the manuscript.

## Conflict of Interest

The authors declare that the research was conducted in the absence of any commercial or financial relationships that could be construed as a potential conflict of interest.

## Footnotes

1 http://www.r-project.org/

2 http://circos.ca/

3 https://gephi.org
