# Supplementary material for: Underlying Mechanism of Wild Radix pseudostellariae in Tolerance to Disease Under the Natural Forest Cover
Source: Front Microbiol. 2020 May 27;11:1142. doi: 10.3389/fmicb.2020.01142 (PMC7266878; doi:10.3389/fmicb.2020.01142)
Supplement: Supplementary file 1 [file Data_Sheet_1.docx]

**Underlying mechanism of wild *Radix pseudostellariae*** **in tolerance to disease under natural forest cover**

Hongmiao Wu^1,2^, Jinshen Xia^1,2^, Xianjin Qin^2,3^, Huiming Wu^1,2^, Shengkai Zhang^1,2^, Yanlin Zhao^1,2^, Christopher Rensing^4*^, Wenxiong Lin^1,2,3*^

^1^ Fujian Provincial Key Laboratory of Agroecological Processing and Safety Monitoring, College of Life Sciences, Fujian Agriculture and Forestry University, Fuzhou 350002, PR China.

^2^ Key Laboratory of Crop Ecology and Molecular Physiology, Fujian Agriculture and Forestry University, Fuzhou 350002, PR China.

^3^ Key Laboratory for Genetics, Breeding and Multiple Utilization of Crops, Ministry of Education / College of Crop Science, Fujian Agriculture and Forestry University, Fuzhou 350002, PR China.

^4^ Fujian Provincial Key Laboratory of Soil Environmental Health and Regulation, College of Resources and Environment, Fujian Agriculture and Forestry University, Fuzhou 350002, PR China.

**Table S1** Primers used for quantitative reverse transcription PCR in this study

| **Primers Name** | **Sequence (5´- 3´)** | **Reference** |
| --- | --- | --- |
| 341F | CCTAYGGGRBGCASCAG | (Ikoyi *et al.*, 2018) |
| 806R | GGACTACNNGGGTATCTAAT |  |
| ITS1F | CTTGGTCATTTAGAGGAAGTAA | (Yao *et al.*, 2017) |
| ITS2R | GCTGCGTTCTTCATCGATGC |  |
| Eub338 | ACTCCTACGGGAGGCAGCAG | (Kielak *et al.*, 2008) |
| Eub518 | ATTACCGCGGCTGCTGG | (T Weedon *et al.*, 2012) |
| ITS1F | CTTGGTCATTTAGAGGAAGTAA | (Onwuchekwa *et al.*, 2014) |
| ITS4 | TCCTCCGCTTATTGATATGC | (Gao *et al.*, 2008, Okubo & Sugiyama, 2009) |
| ITS1F | CTTGGTCATTTAGAGGA AGTAA | (Lievens *et al.*, 2005) |
| AFP308R | CGAATTAACGCGAGTCCCAA |  |
| FolSSR-3F | CTCGCATACTACTACCGCACAG | (Mahfooz *et al.*, 2012) |
| FolSSR-3R | GCAGATAAGGGAGATGCAAAAC |  |

**Table S2** Statistical table of microbial groups at each classification level.

| Treatments | Kingdom | Phylum | Class | Order | Family | Genus |
| --- | --- | --- | --- | --- | --- | --- |
| chu2 | Fungi | 10a | 21b | 69a | 116a | 192b |
| chu4 |  | 9b | 20b | 66b | 115a | 187b |
| chu3 |  | 9b | 22ab | 68a | 118a | 202a |
| chu5 |  | 10a | 24a | 66b | 112a | 185b |
| chu2 | Bacteria | 21a | 72a | 105a | 189a | 265a |
| chu4 |  | 20a | 72a | 92b | 162c | 205c |
| chu3 |  | 21a | 73a | 100a | 169bc | 210c |
| chu5 |  | 21a | 73a | 102a | 175b | 236b |

The different letters in each column represent significant differences (LSD-test, *p* <0.05, n = 3).

**Table S3** Relative abundance of the microbial genus among the different samples

| Genus | Family | Order | Class | Phylum | Relative abundance (%) | | | |
| --- | --- | --- | --- | --- | --- | --- | --- | --- |
|  |  |  |  |  | chu2 | chu4 | chu3 | chu5 |
| *Penicillium* | *Trichocomaceae* | *Eurotiales* | *Eurotiomycetes* | Ascomycota | 0.2186 | 1.0042 | 0.2377 | 0.2196 |
| *Aspergillus* | *Trichocomaceae* | *Eurotiales* | *Eurotiomycetes* | Ascomycota | 0.1170 | 0.0516 | 0.7310 | 0.3558 |
| *Fusarium* | *Nectriaceae* | *Hypocreales* | *Sordariomycetes* | Ascomycota | 0.2869 | 0.0683 | 0.2432 | 0.2738 |
| *Nitrobacter* | *Bradyrhizobiaceae* | *Rhizobiales* | *Alphaproteobacteria* | Proteobacteria | 0.8225 | 1.2732 | 0.2906 | 0.6162 |
| *Nitrospira* | *Nitrospiraceae* | *Nitrospirales* | *Nitrospira* | Nitrospirae | 0.3335 | 0.2068 | 0.3830 | 0.4090 |
| *Streptomyces* | *Streptomycetaceae* | *Streptomycetales* | *Actinobacteria* | Actinobacteria | 0.4001 | 0.2238 | 0.1440 | 0.2256 |
| *Actinoplanes* | *Micromonosporaceae* | *Micromonosporales* | *Actinobacteria* | Actinobacteria | 0.1917 | 0.1853 | 0.2716 | 0.2293 |
| *Pseudomonas* | *Pseudomonadaceae* | *Pseudomonadales* | *Gammaproteobacteria* | Proteobacteria | 0.1250 | 0.0081 | 0.0407 | 0.1354 |

**Table S4** Venn diagram statistics of predominantly shared and exclusive genus-level taxa of fungi across all the samples.

| **Venn groups** | **Phylum** | **Class** | **Order** | **Family** | **Genus** | **OTUs**  **numbers** |
| --- | --- | --- | --- | --- | --- | --- |
| **Shared** | Basidiomycota | Tremellomycetes | *Tremellales* | *Incertae sedis* | *Bullera* | 5 |
|  | Ascomycota | Lecanoromycetes | *Peltigerales* | *Pannariaceae* | *Pectenia* | 4 |
|  | Ascomycota | Eurotiomycetes | *Eurotiales* | *Trichocomaceae* | *Penicillium* | 4 |
|  | Ascomycota | Eurotiomycetes | *Chaetothyriales* | *Herpotrichiellaceae* | *Cladophialophora* | 3 |
|  | Ascomycota | Dothideomycetes | *Capnodiales* | *Teratosphaeriaceae* | *Devriesia* | 3 |
|  | Ascomycota | Sordariomycetes | *Hypocreales* | *Nectriaceae* | *Fusarium* | 3 |
|  | Zygomycota | Incertae sedis | *Mortierellales* | *Mortierellaceae* | *Mortierella* | 3 |
|  | Ascomycota | Sordariomycetes | *Sordariales* | *Lasiosphaeriaceae* | *Podospora* | 3 |
| **Exclusive chu2** | Basidiomycota | Tremellomycetes | *Tremellales* | *Incertae sedis* | *Bullera* | 34 |
|  | Zygomycota | Incertae sedis | *Basidiobolales* | *Basidiobolaceae* | *Basidiobolus* | 13 |
|  | Ascomycota | Saccharomycetes | *Saccharomycetales* | *Incertae sedis* | *Candida* | 6 |
|  | Basidiomycota | Tremellomycetes | *Tremellales* | *Incertae sedis* | *Bulleribasidium* | 4 |
|  | Basidiomycota | Agaricomycetes | *Boletales* | *Boletaceae* | *Octaviania* | 4 |
|  | Basidiomycota | Agaricomycetes | *Agaricales* | *Inocybaceae* | *Inocybe* | 3 |
|  | Zygomycota | Incertae sedis | *Mortierellales* | *Mortierellaceae* | *Mortierella* | 3 |
|  | Basidiomycota | Agaricomycetes | *Russulales* | *Russulaceae* | *Russula* | 3 |
| **Exclusive chu4** | Basidiomycota | Tremellomycetes | *Tremellales* | *Incertae sedis* | *Bullera* | 20 |
|  | Basidiomycota | Agaricomycetes | *Thelephorales* | *Thelephoraceae* | *Tomentella* | 12 |
|  | Zygomycota | Incertae sedis | *Basidiobolales* | *Basidiobolaceae* | *Basidiobolus* | 7 |
|  | Basidiomycota | Agaricomycetes | *Sebacinales* | *Sebacinaceae* | *Sebacina* | 7 |
|  | Ascomycota | Eurotiomycetes | *Eurotiales* | *Trichocomaceae* | *Penicillium* | 5 |
|  | Ascomycota | Sordariomycetes | *Sordariales* | *Lasiosphaeriaceae* | *Podospora* | 5 |
|  | Basidiomycota | Agaricomycetes | *Cantharellales* | *Clavulinaceae* | *Clavulina* | 3 |
|  | Basidiomycota | Agaricomycetes | *Agaricales* | *Entolomataceae* | *Entoloma* | 3 |
|  | Zygomycota | Incertae sedis | *Mortierellales* | *Mortierellaceae* | *Mortierella* | 3 |
|  | Basidiomycota | Agaricomycetes | *Boletales* | *Sclerodermataceae* | *Scleroderma* | 3 |
| **Exclusive chu3** | Basidiomycota | Tremellomycetes | *Tremellales* | *Incertae sedis* | *Bullera* | 36 |
|  | Ascomycota | Eurotiomycetes | *Eurotiales* | *Trichocomaceae* | *Penicillium* | 6 |
|  | Zygomycota | Incertae sedis | *Basidiobolales* | *Basidiobolaceae* | *Basidiobolus* | 5 |
|  | Zygomycota | Incertae sedis | *Mortierellales* | *Mortierellaceae* | *Mortierella* | 5 |
|  | Basidiomycota | Agaricomycetes | *Agaricales* | *Psathyrellaceae* | *Coprinopsis* | 4 |
|  | Basidiomycota | Agaricomycetes | *Agaricales* | *Pluteaceae* | *Pluteus* | 4 |
|  | Ascomycota | Pezizomycetes | *Pezizales* | *Tuberaceae* | *Tuber* | 4 |
|  | Ascomycota | Eurotiomycetes | *Eurotiales* | *Trichocomaceae* | *Aspergillus* | 3 |
|  | Basidiomycota | Agaricomycetes | *Agaricales* | *Entolomataceae* | *Entoloma* | 3 |
|  | Ascomycota | Pezizomycetes | *Pezizales* | *Discinaceae* | *Hydnotrya* | 3 |
|  | Basidiomycota | Microbotryomycetes | *Sporidiobolales* | *Incertae sedis* | *Rhodotorula* | 3 |
|  | Zygomycota | Incertae sedis | *Basidiobolales* | *Basidiobolaceae* | *Schizangiella* | 3 |
| **Exclusive chu5** | Basidiomycota | Tremellomycetes | *Tremellales* | *Incertae sedis* | *Bullera* | 24 |
|  | Ascomycota | Archaeorhizomycetes | *Archaeorhizomycetales* | *Archaeorhizomycetaceae* | *Archaeorhizomyces* | 4 |
|  | Ascomycota | Incertae sedis | *Incertae sedis* | *Incertae sedis* | *Chalara* | 3 |
|  | Zygomycota | Incertae sedis | *Mortierellales* | *Mortierellaceae* | *Mortierella* | 3 |
|  | Ascomycota | Dothideomycetes | *Incertae sedis* | *Incertae sedis* | *Peltaster* | 3 |

**Table S5** Venn diagram statistics of predominantly shared and exclusive genus-level taxa of bacteria across all the samples.

| **Venn groups** | **Phylum** | **Class** | **Order** | **Family** | **Genus** | **OTUs**  **numbers** |
| --- | --- | --- | --- | --- | --- | --- |
| **Shared** | Proteobacteria | Deltaproteobacteria | *Myxococcales* | *Haliangiaceae* | *Haliangium* | 21 |
|  | Acidobacteria | Blastocatellia | *Blastocatellales* | *Blastocatellaceae* | *RB41* | 18 |
|  | Acidobacteria | Solibacteres | *Solibacterales* | *Solibacteraceae* | *Bryobacter* | 17 |
|  | Gemmatimonadetes | Gemmatimonadetes | *Gemmatimonadales* | *Gemmatimonadaceae* | *Gemmatimonas* | 13 |
|  | Acidobacteria | Solibacteres | *Solibacterales* | *Solibacteraceae* | *Candidatus_Solibacter* | 12 |
|  | Actinobacteria | Actinobacteria | *Propionibacteriales* | *Nocardioidaceae* | *Nocardioides* | 11 |
|  | Proteobacteria | Deltaproteobacteria | *Desulfurellales* | *Desulfurellaceae* | *H16* | 10 |
|  | Chloroflexi | Chloroflexia | *Chloroflexales* | *Roseiflexaceae* | *Roseiflexus* | 10 |
|  | Actinobacteria | Thermoleophilia | *Solirubrobacterales* | *Solirubrobacteraceae* | *Solirubrobacter* | 10 |
|  | Acidobacteria | Solibacteres | *Solibacterales* | *Solibacteraceae* | *Paludibaculum* | 9 |
|  | Proteobacteria | Betaproteobacteria | *--* | *--* | *--* | 8 |
|  | Proteobacteria | Alphaproteobacteria | *Rhizobiales* | *Xanthobacteraceae* | *Variibacter* | 8 |
|  | Acidobacteria | Blastocatellia | *Blastocatellales* | *Blastocatellaceae* | *11-24* | 7 |
|  | Actinobacteria | Acidimicrobiia | *Acidimicrobiales* | *Iamiaceae* | *Iamia* | 7 |
|  | Actinobacteria | Actinobacteria | *Corynebacteriales* | *Mycobacteriaceae* | *Mycobacterium* | 7 |
|  | Proteobacteria | Alphaproteobacteria | *Rhizobiales* | *Bradyrhizobiaceae* | *Nitrobacter* | 6 |
|  | Actinobacteria | Actinobacteria | *Pseudonocardiales* | *Pseudonocardiaceae* | *Pseudonocardia* | 6 |
|  | Planctomycetes | Phycisphaerae | *Phycisphaerales* | *Phycisphaeraceae* | *SM1A02* | 6 |
|  | Proteobacteria | Alphaproteobacteria | *Sphingomonadales* | *Sphingomonadaceae* | *Sphingomonas* | 6 |
|  | Planctomycetes | Phycisphaerae | *Phycisphaerales* | *Phycisphaeraceae* | *AKYG587* | 5 |
|  | Actinobacteria | Acidimicrobiia | *Acidimicrobiales* | *Acidimicrobiaceae* | *CL500-29* | 5 |
|  | Proteobacteria | Gammaproteobacteria | *Xanthomonadales* | *Xanthomonadales* | *Acidibacter* | 4 |
|  | Actinobacteria | Actinobacteria | *Micromonosporales* | *Micromonosporaceae* | *Actinoplanes* | 4 |
|  | Bacteroidetes | Flavobacteriia | *Flavobacteriales* | *Flavobacteriaceae* | *Flavobacterium* | 4 |
|  | Actinobacteria | Thermoleophilia | *Gaiellales* | *Gaiellaceae* | *Gaiella* | 4 |
|  | Proteobacteria | Deltaproteobacteria | *Bdellovibrionales* | *Bdellovibrionaceae* | *OM27* | 4 |
|  | Proteobacteria | Alphaproteobacteria | *Rhizobiales* | *Hyphomicrobiaceae* | *Rhodoplanes* | 4 |
|  | Proteobacteria | Deltaproteobacteria | *Myxococcales* | *Polyangiaceae* | *Sorangium* | 4 |
|  | Actinobacteria | Actinobacteria | *Streptomycetales* | *Streptomycetaceae* | *Streptomyces* | 4 |
| **Exclusive chu2** | Chloroflexi | Chloroflexia | *Chloroflexales* | *Roseiflexaceae* | *Roseiflexus* | 20 |
|  | Proteobacteria | Deltaproteobacteria | *Myxococcales* | *Haliangiaceae* | *Haliangium* | 15 |
|  | Bacteroidetes | Flavobacteriia | *Flavobacteriales* | *Flavobacteriaceae* | *Flavobacterium* | 12 |
|  | Proteobacteria | Deltaproteobacteria | *Bdellovibrionales* | *Bdellovibrionaceae* | *Bdellovibrio* | 11 |
|  | Gemmatimonadetes | Gemmatimonadetes | *Gemmatimonadales* | *Gemmatimonadaceae* | *Gemmatimonas* | 11 |
|  | Planctomycetes | Phycisphaerae | *Phycisphaerales* | *Phycisphaeraceae* | *SM1A02* | 11 |
|  | Actinobacteria | Actinobacteria | *Propionibacteriales* | *Nocardioidaceae* | *Nocardioides* | 9 |
|  | Bacteroidetes | Sphingobacteriia | *Sphingobacteriales* | *Chitinophagaceae* | *Chitinophaga* | 8 |
|  | Proteobacteria | Alphaproteobacteria | *Sphingomonadales* | *Sphingomonadaceae* | *Sphingomonas* | 8 |
|  | Acidobacteria | Blastocatellia | *Blastocatellales* | *Blastocatellaceae* | *11-24* | 7 |
|  | Proteobacteria | Deltaproteobacteria | *Myxococcales* | *Polyangiaceae* | *Sorangium* | 7 |
|  | Verrucomicrobia | Spartobacteria | *Chthoniobacterales* | *Chthoniobacteraceae* | *Chthoniobacter* | 6 |
|  | Actinobacteria | Actinobacteria | *Micromonosporales* | *Micromonosporaceae* | *Luedemannella* | 6 |
|  | Proteobacteria | Deltaproteobacteria | *Myxococcales* | *Phaselicystidaceae* | *Phaselicystis* | 6 |
|  | Acidobacteria | Blastocatellia | *Blastocatellales* | *Blastocatellaceae* | *RB41* | 6 |
|  | Proteobacteria | Alphaproteobacteria | *Rhizobiales* | *Bradyrhizobiaceae* | *Bradyrhizobium* | 5 |
|  | Proteobacteria | Gammaproteobacteria | *Enterobacteriales* | *Enterobacteriaceae* | *Escherichia-Shigella* | 5 |
|  | Proteobacteria | Gammaproteobacteria | *Xanthomonadales* | *Xanthomonadaceae* | *Arenimonas* | 4 |
|  | Acidobacteria | Solibacteres | *Solibacterales* | *Solibacteraceae* | *Bryobacter* | 4 |
|  | Proteobacteria | Deltaproteobacteria | *Desulfurellales* | *Desulfurellaceae* | *H16* | 4 |
|  | Actinobacteria | Acidimicrobiia | *Acidimicrobiales* | *Iamiaceae* | *Iamia* | 4 |
|  | Proteobacteria | Deltaproteobacteria | *Myxococcales* | *Polyangiaceae* | *Polyangium* | 4 |
|  | Proteobacteria | Alphaproteobacteria | *Rhodospirillales* | *Acetobacteraceae* | *Roseomonas* | 4 |
|  | Proteobacteria | Alphaproteobacteria | *Caulobacterales* | *Hyphomonadaceae* | *Woodsholea* | 4 |
| **Exclusive chu4** | Actinobacteria | Actinobacteria | *Frankiales* | *Acidothermaceae* | *Acidothermus* | 57 |
|  | Gemmatimonadetes | Gemmatimonadetes | *Gemmatimonadales* | *Gemmatimonadaceae* | *Gemmatimonas* | 35 |
|  | Acidobacteria | Solibacteres | *Solibacterales* | *Solibacteraceae_Subgroup3* | *Candidatus_Solibacter* | 29 |
|  | Acidobacteria | Solibacteres | *Solibacterales* | *Solibacteraceae_Subgroup3* | *Bryobacter* | 25 |
|  | Proteobacteria | Deltaproteobacteria | *Myxococcales* | *Haliangiaceae* | *Haliangium* | 23 |
|  | Proteobacteria | Alphaproteobacteria | *Rhizobiales* | *Xanthobacteraceae* | *Variibacter* | 18 |
|  | Proteobacteria | Alphaproteobacteria | *Rhizobiales* | *Rhizobiales_Incertae Sedis* | *Rhizomicrobium* | 15 |
|  | Acidobacteria | Blastocatellia | *Blastocatellales* | *Blastocatellaceae* | *RB41* | 14 |
|  | Proteobacteria | Deltaproteobacteria | *Desulfurellales* | *Desulfurellaceae* | *H16* | 11 |
|  | Planctomycetes | Planctomycetacia | *Planctomycetales* | *Planctomycetaceae* | *Singulisphaera* | 11 |
|  | Proteobacteria | Alphaproteobacteria | *Sphingomonadales* | *Sphingomonadaceae* | *Sphingomonas* | 11 |
|  | Proteobacteria | Gammaproteobacteria | *Xanthomonadales* | *Xanthomonadales_Incertae Sedis* | *Acidibacter* | 10 |
|  | Chloroflexi | Chloroflexia | *Chloroflexales* | *Roseiflexaceae* | *Roseiflexus* | 10 |
|  | Acidobacteria | Blastocatellia | *Blastocatellales* | *Blastocatellaceae_Subgroup3* | *11-24* | 8 |
|  | Actinobacteria | Actinobacteria | *Frankiales* | *Frankiaceae* | *Jatrophihabitans* | 8 |
|  | Proteobacteria | Betaproteobacteria | *Burkholderiales* | *Burkholderiaceae* | *Burkholderia-Paraburkholderia* | 7 |
|  | Proteobacteria | Alphaproteobacteria | *Rhizobiales* | *Xanthobacteraceae* | *Pseudolabrys* | 6 |
|  | Proteobacteria | Deltaproteobacteria | *Myxococcales* | *Polyangiaceae* | *Sorangium* | 6 |
|  | Actinobacteria | Actinobacteria | *Streptosporangiales* | *Thermomonosporaceae* | *Actinomadura* | 5 |
|  | Proteobacteria | Gammaproteobacteria | *Legionellales* | *Coxiellaceae* | *Aquicella* | 5 |
|  | Gemmatimonadetes | Gemmatimonadetes | *Gemmatimonadales* | *Gemmatimonadaceae* | *Gemmatirosa* | 5 |
|  | Chloroflexi | Ktedonobacteria | *Ktedonobacterales* | *Ktedonobacteraceae* | *Ktedonobacter* | 5 |
|  | Actinobacteria | Actinobacteria | *Micromonosporales* | *Micromonosporaceae* | *Luedemannella* | 5 |
|  | Actinobacteria | Actinobacteria | *Micromonosporales* | *Micromonosporaceae* | *Actinoplanes* | 4 |
|  | Actinobacteria | Actinobacteria | *Micromonosporales* | *Micromonosporaceae* | *Asanoa* | 4 |
|  | Proteobacteria | Alphaproteobacteria | *Rhizobiales* | *Bradyrhizobiaceae* | *Bradyrhizobium* | 4 |
|  | Actinobacteria | Actinobacteria | *Corynebacteriales* | *Mycobacteriaceae* | *Mycobacterium* | 4 |
| **Exclusive chu3** | Proteobacteria | Deltaproteobacteria | *Myxococcales* | *Haliangiaceae* | *Haliangium* | 19 |
|  | Acidobacteria | Blastocatellia | *Blastocatellales* | *Blastocatellaceae* | *RB41* | 11 |
|  | Proteobacteria | Deltaproteobacteria | *Desulfurellales* | *Desulfurellaceae* | *H16* | 10 |
|  | Chloroflexi | Chloroflexia | *Chloroflexales* | *Roseiflexaceae* | *Roseiflexus* | 9 |
|  | Verrucomicrobia | Spartobacteria | *Chthoniobacterales* | *Chthoniobacteraceae* | *Chthoniobacter* | 7 |
|  | Acidobacteria | Blastocatellia | *Blastocatellales* | *Blastocatellaceae* | *(Subgroup* | 4 |
|  | Planctomycetes | Phycisphaerae | *Phycisphaerales* | *Phycisphaeraceae* | *AKYG587* | 4 |
|  | Acidobacteria | Solibacteres | *Solibacterales* | *Solibacteraceae* | *Candidatus_Solibacter* | 4 |
|  | Bacteroidetes | Flavobacteriia | *Flavobacteriales* | *Flavobacteriaceae* | *Flavobacterium* | 4 |
|  | Planctomycetes | Phycisphaerae | *Phycisphaerales* | *Phycisphaeraceae* | *SM1A02* | 4 |
|  | Actinobacteria | Acidimicrobiia | *Acidimicrobiales* | *uncultured* | *Cellulomonadaceae* | 4 |
| **Exclusive chu5** | Proteobacteria | Deltaproteobacteria | *Myxococcales* | *Haliangiaceae* | *Haliangium* | 14 |
|  | Acidobacteria | Blastocatellia | *Blastocatellales* | *Blastocatellaceae_Subgroup4* | *11-24* | 7 |
|  | Acidobacteria | Solibacteres | *Solibacterales* | *Solibacteraceae_Subgroup3* | *Bryobacter* | 7 |
|  | Acidobacteria | Holophagae | *Subgroup10* | *ABS-19* | *Bryobacter* | 7 |
|  | Acidobacteria | Solibacteres | *Solibacterales* | *Solibacteraceae* | *Candidatus_Solibacter* | 6 |
|  | Chloroflexi | Chloroflexia | *Chloroflexales* | *Roseiflexaceae* | *Roseiflexus* | 5 |
|  | Planctomycetes | Phycisphaerae | *Phycisphaerales* | *Phycisphaeraceae* | *SM1A02* | 5 |
|  | Acidobacteria | Blastocatellia | *Blastocatellales* | *Blastocatellaceae* | *Elev-16S-573* | 4 |
|  | Proteobacteria | Deltaproteobacteria | *Desulfurellales* | *Desulfurellaceae* | *H16* | 4 |
|  | Actinobacteria | Actinobacteria | *Micromonosporales* | *Micromonosporaceae* | *Luedemannella* | 4 |
|  | Firmicutes | Clostridia | *Clostridiales* | *Family_XIII* | *Mogibacterium* | 4 |
|  | Proteobacteria | Deltaproteobacteria | *Myxococcales* | *Polyangiaceae* | *Sorangium* | 4 |

**Table S6** Network properties of soil bacterial and fungal communities.

| Classified | Treatments | Average degree  (AD) | Network  diameter (ND) | Modularity  (MD) | Clustering  coefficient (CC) | Average path length  (APL) | Nodes | Edges |  |
| --- | --- | --- | --- | --- | --- | --- | --- | --- | --- |
| Fungi | All soil samples | 17.624 | 5 | 1.358 | 0.601 | 2.335 | 101 | 890 | |
| Bacteria | All soil samples | 27.814 | 6 | 6.135 | 0.596 | 2.352 | 161 | 2239 | |

**Table S****7** Analysis of statistical relationship between soil microbial communities and their various soil attributes

|  | Whole data Bray-Curtis distances | | | |
| --- | --- | --- | --- | --- |
| Factor | Fungal ADONIS | | Bacterial ADONIS | |
|  | R^2^ | *p* | R^2^ | *p* |
| NO_3_^−^ -N | 0.313 | 0.183 | 0.6097 | 0.016 * |
| NH_4_^+^-N | 0.2766 | 0.264 | 0.3606 | 0.144 |
| Chitinase | 0.9234 | 0.001 *** | 0.5843 | 0.027 * |
| Cellulase | 0.8776 | 0.001 *** | 0.9282 | 0.001 *** |
| Sucrase | 0.4648 | 0.062 | 0.7642 | 0.002 ** |
| pH | 0.8739 | 0.001 *** | 0.9137 | 0.001 *** |

Note: Significant analysis based on 999 times permutation test; bold *p* values indicate significant difference (*p* < 0.05)


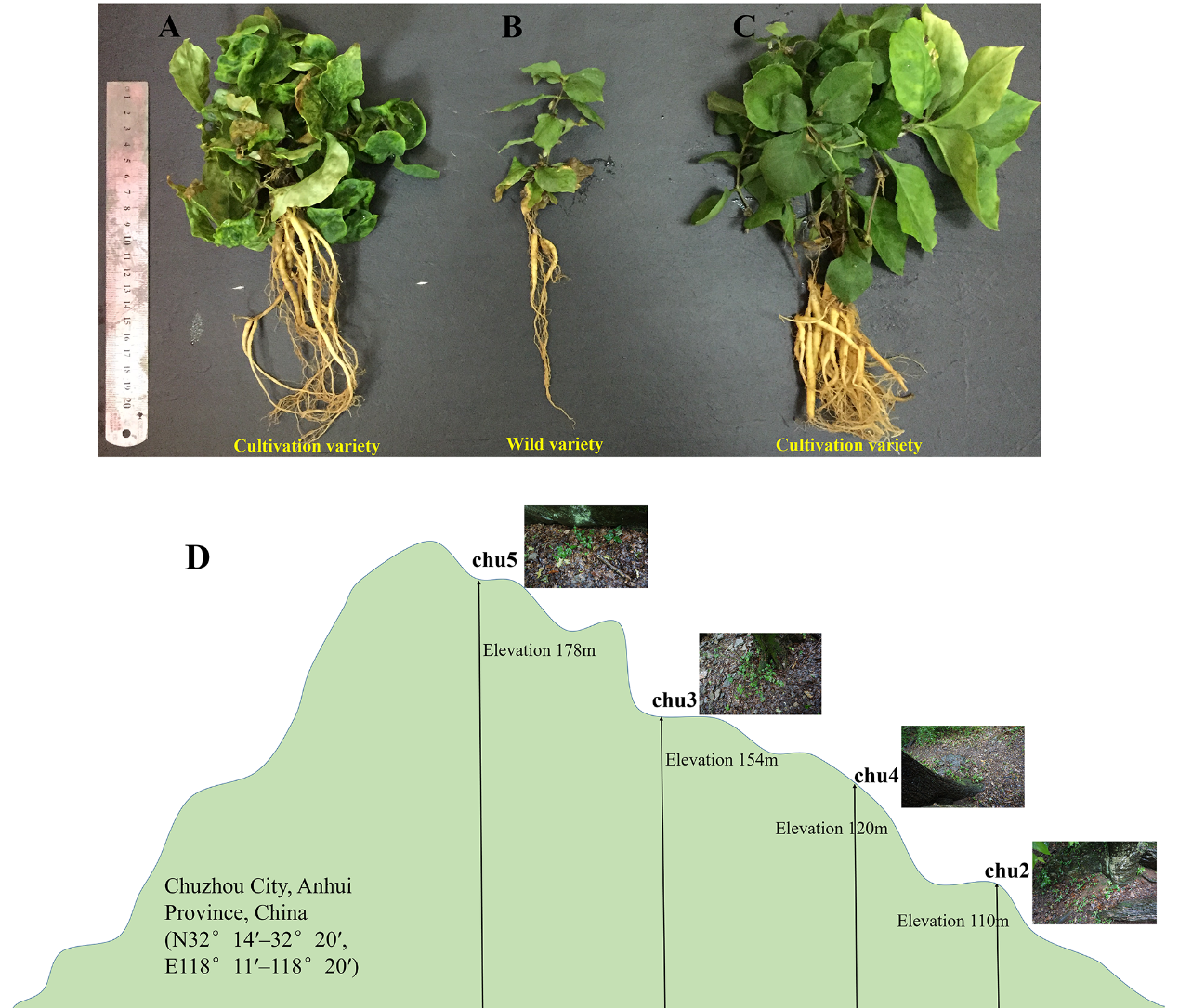


**Figure S1** The different *R. pseudostellariae* cultivars in the two-year consecutively monocultured plots (A, B and C) and sampling sites of the wild medicinal plants under forest in Anhui Provence (D). **A and C** represent the Zheshen 2 and Shitai 1 *R. pseudostellariae* cultivars, respectively. **B** represents the wild *R. pseudostellariae* plants.


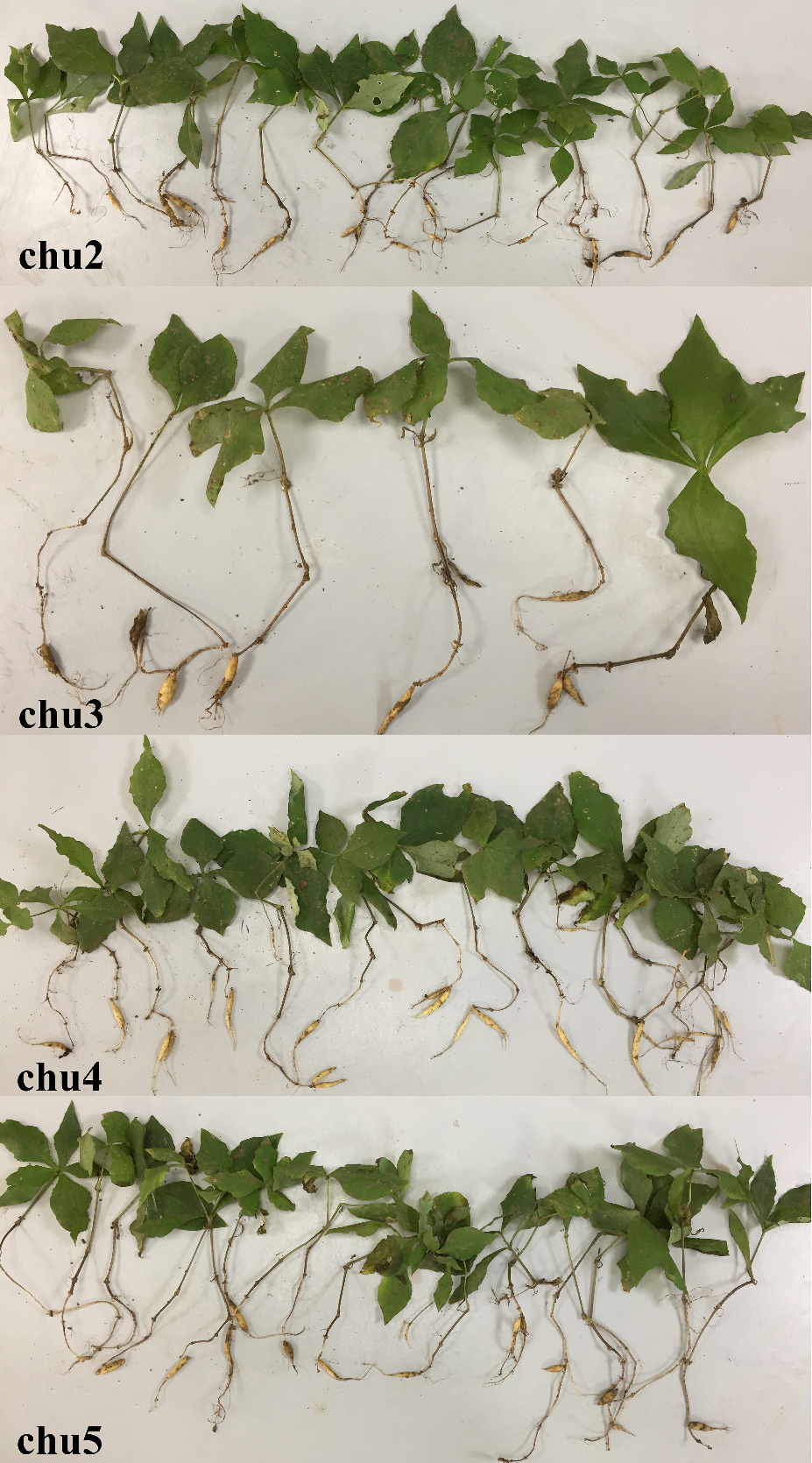


**Figure S2** Growth status and population quantity of *R. pseudostellariae* in different sampling sites.


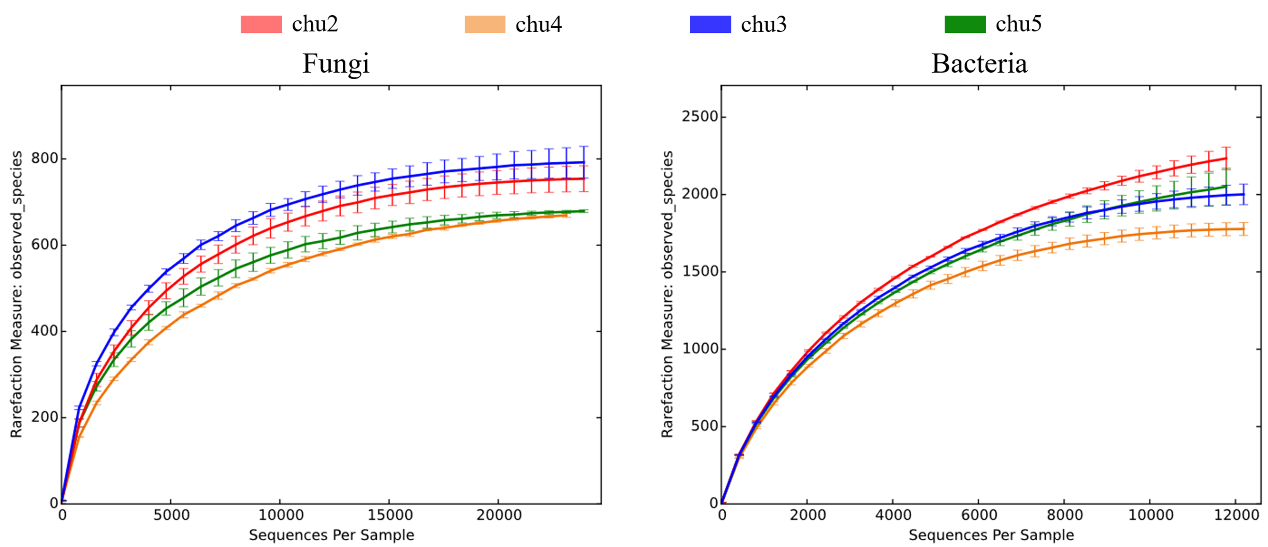


**Figure S3** Rarefaction curves of microbial communities based on observed OTUs at 97% sequence similarity for individual samples.


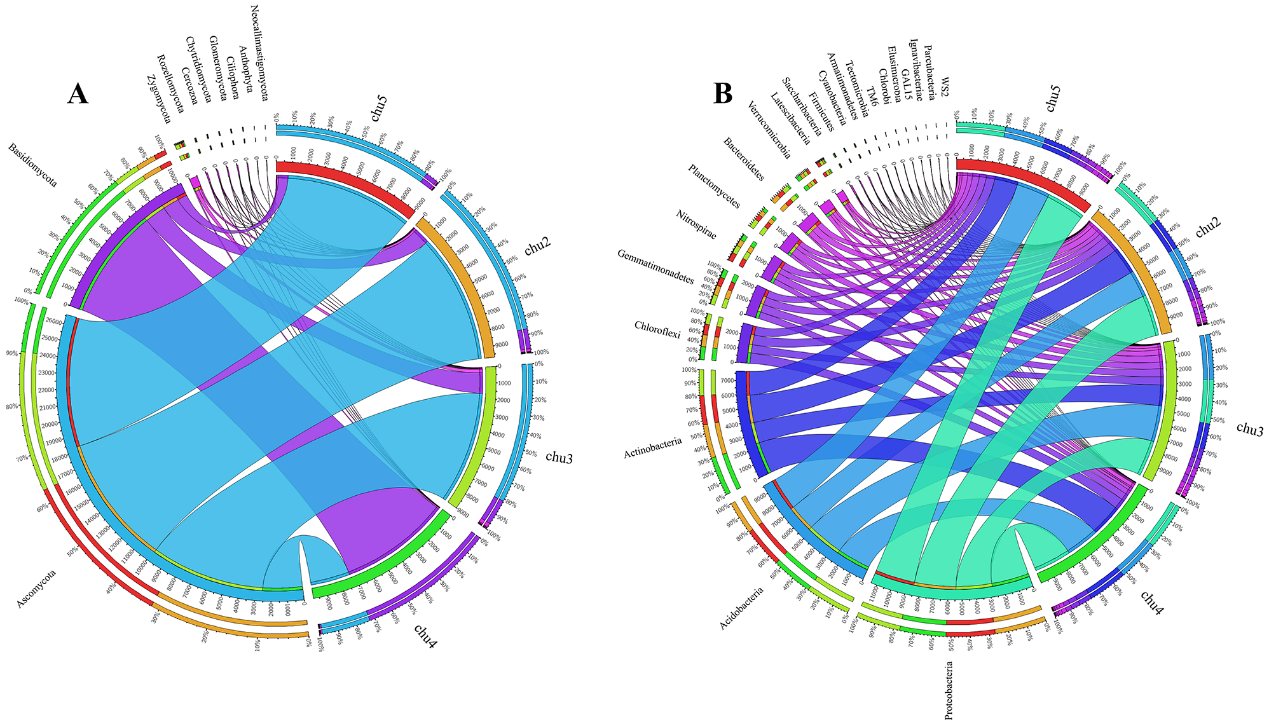


**Figure S4** Distribution of phyla in the whole community in different treatment samples. **A and B**: CIRCOS plots showing the distribution of links among the soil fungal and bacterial phyla under different cultivation patterns. The thickness of each ribbon represents the abundance of each taxon. The absolute tick above the inner segment and relative tick above the outer segment represent the abundance of reads and relative abundance of each taxon, respectively. The data were visualized using Circos (Version 0.69, http://circos.ca/).


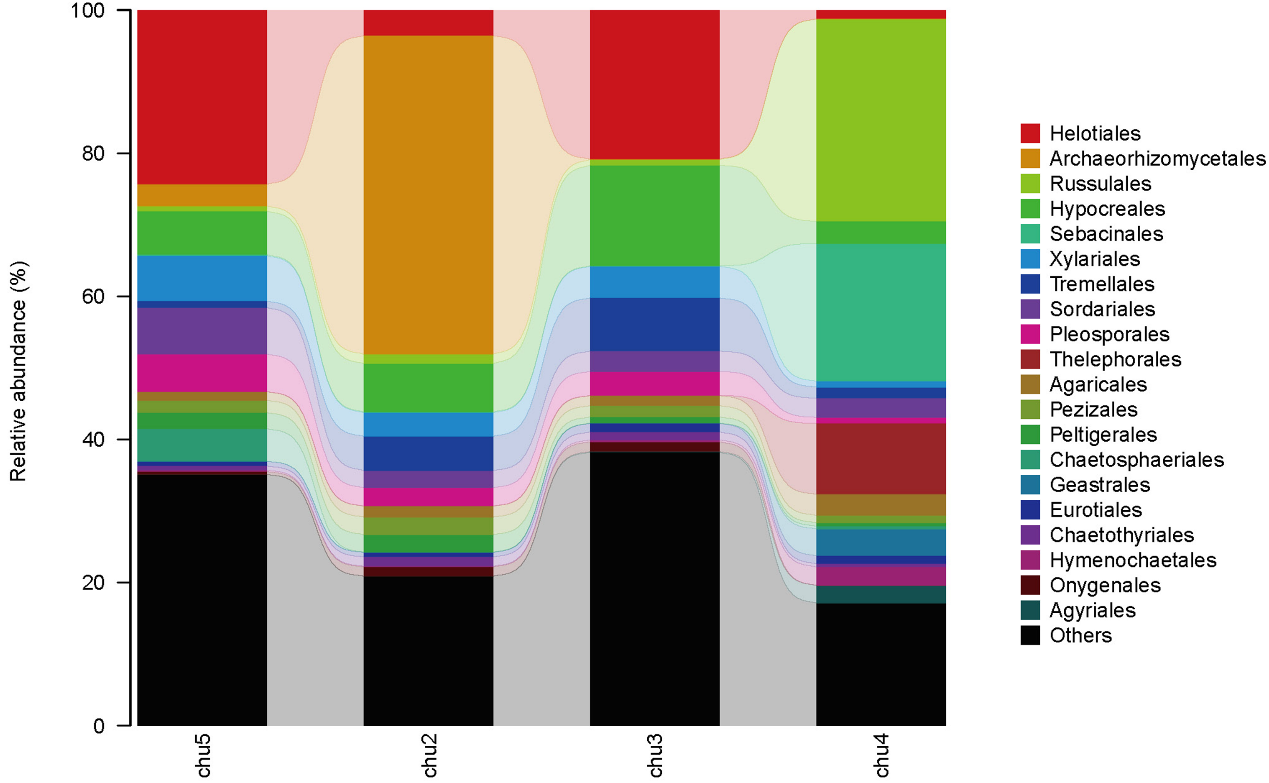


**Figure S5** Relative abundance of the predominant fungal order levels in different samples.


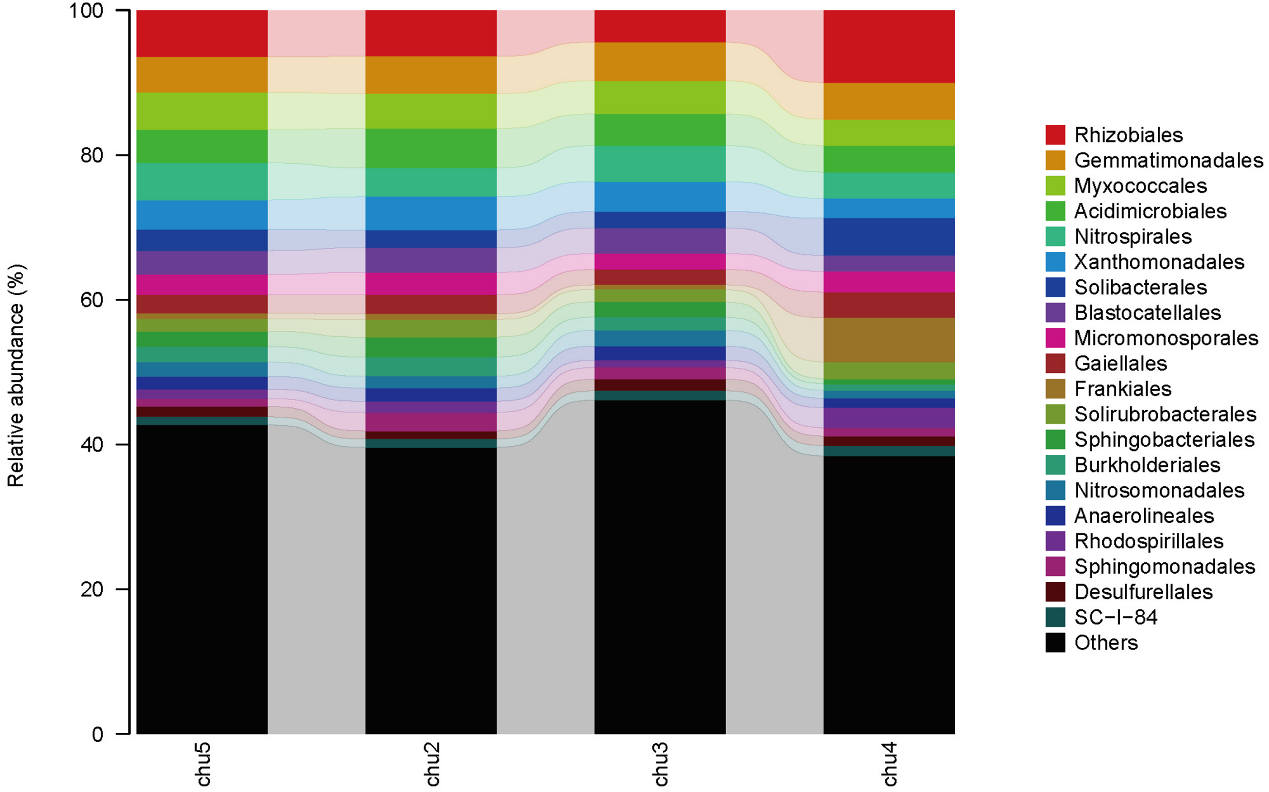


**Figure S6** Relative abundance of the predominant bacterial order levels in different samples.


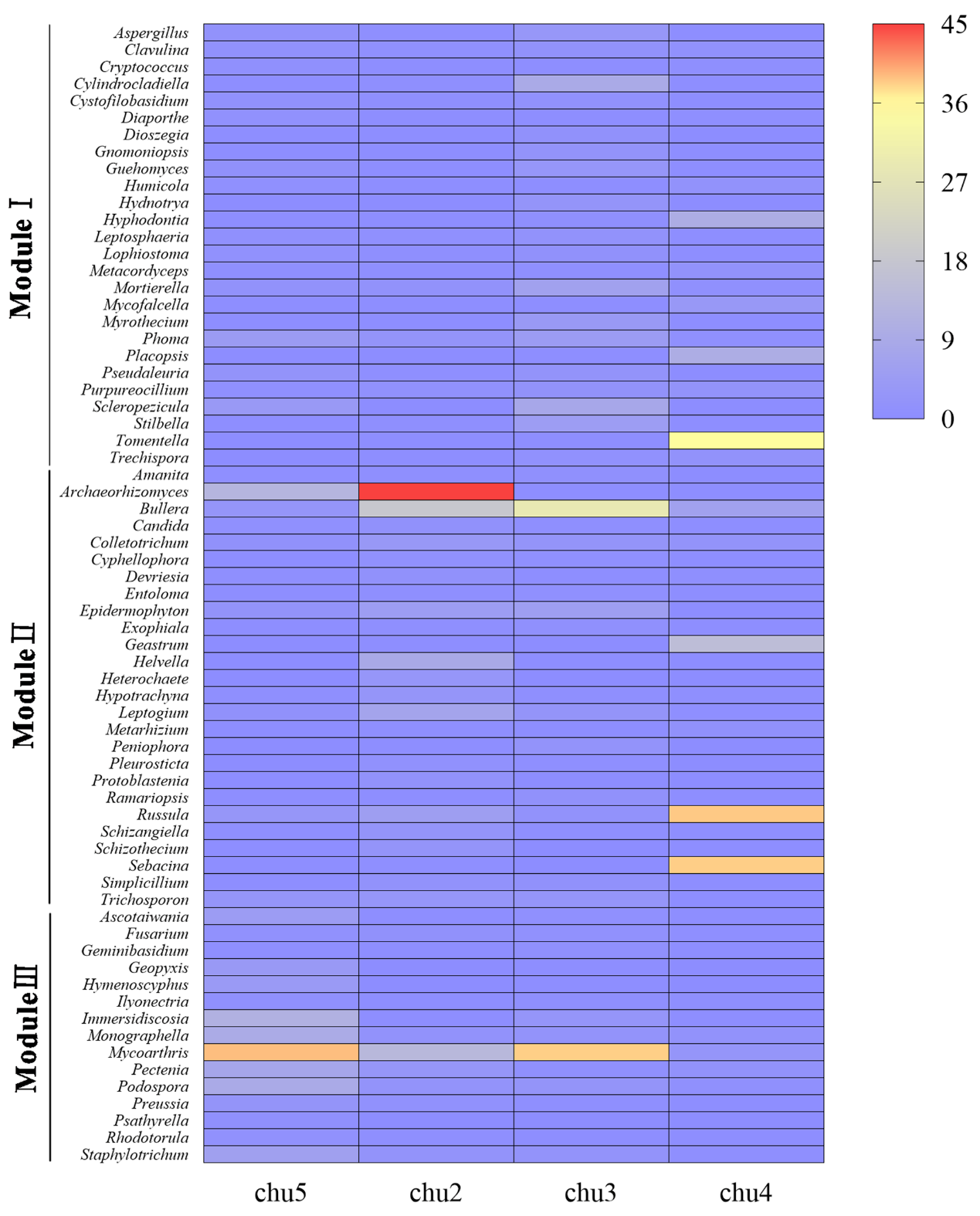


**Figure S7** Relative abundance (%) of the predominantly fungal genus level of modules in fungi.


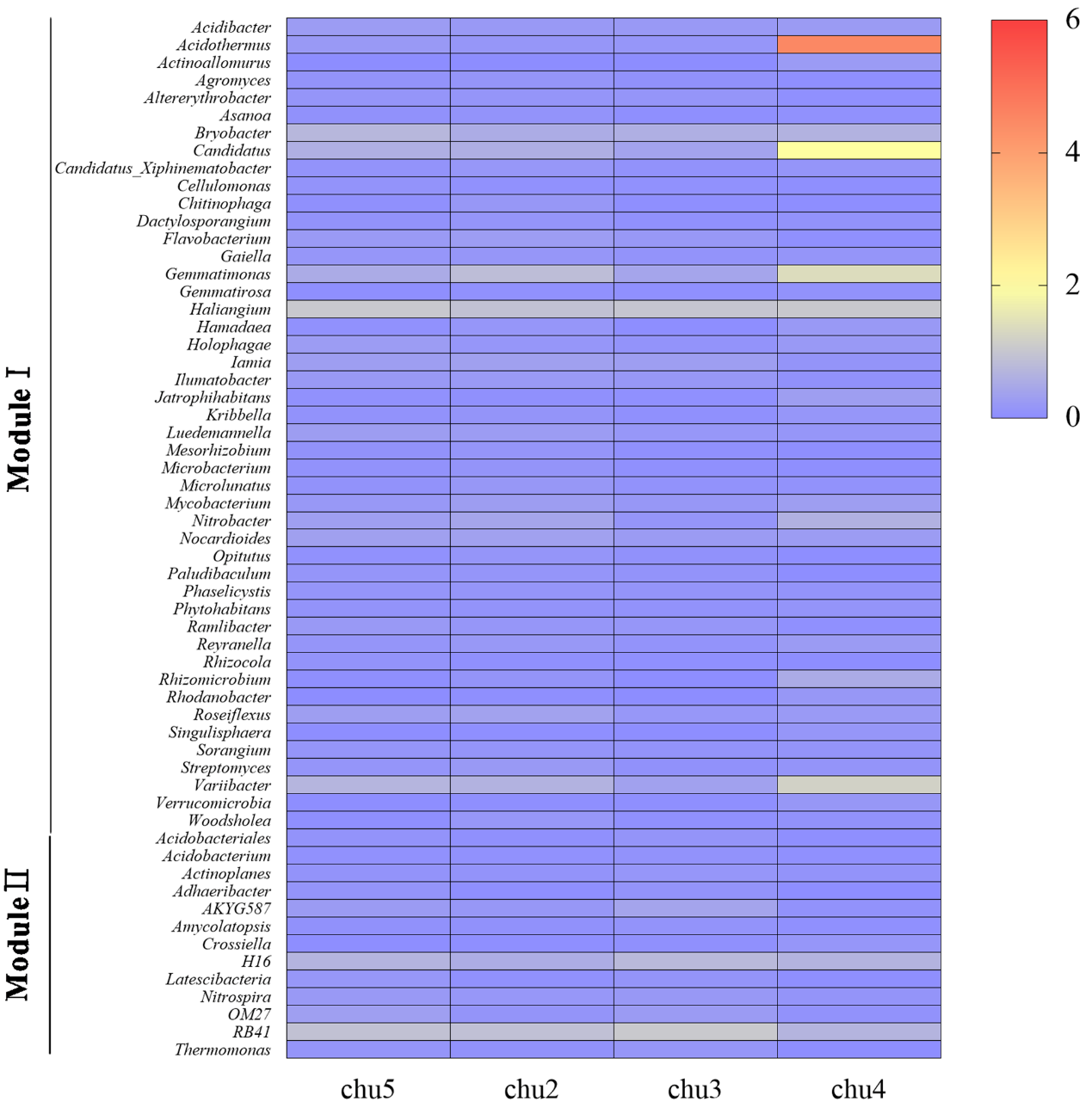


**Figure S8** Relative abundance (%) of the predominantly bacterial genus level of modules in bacteria.

**
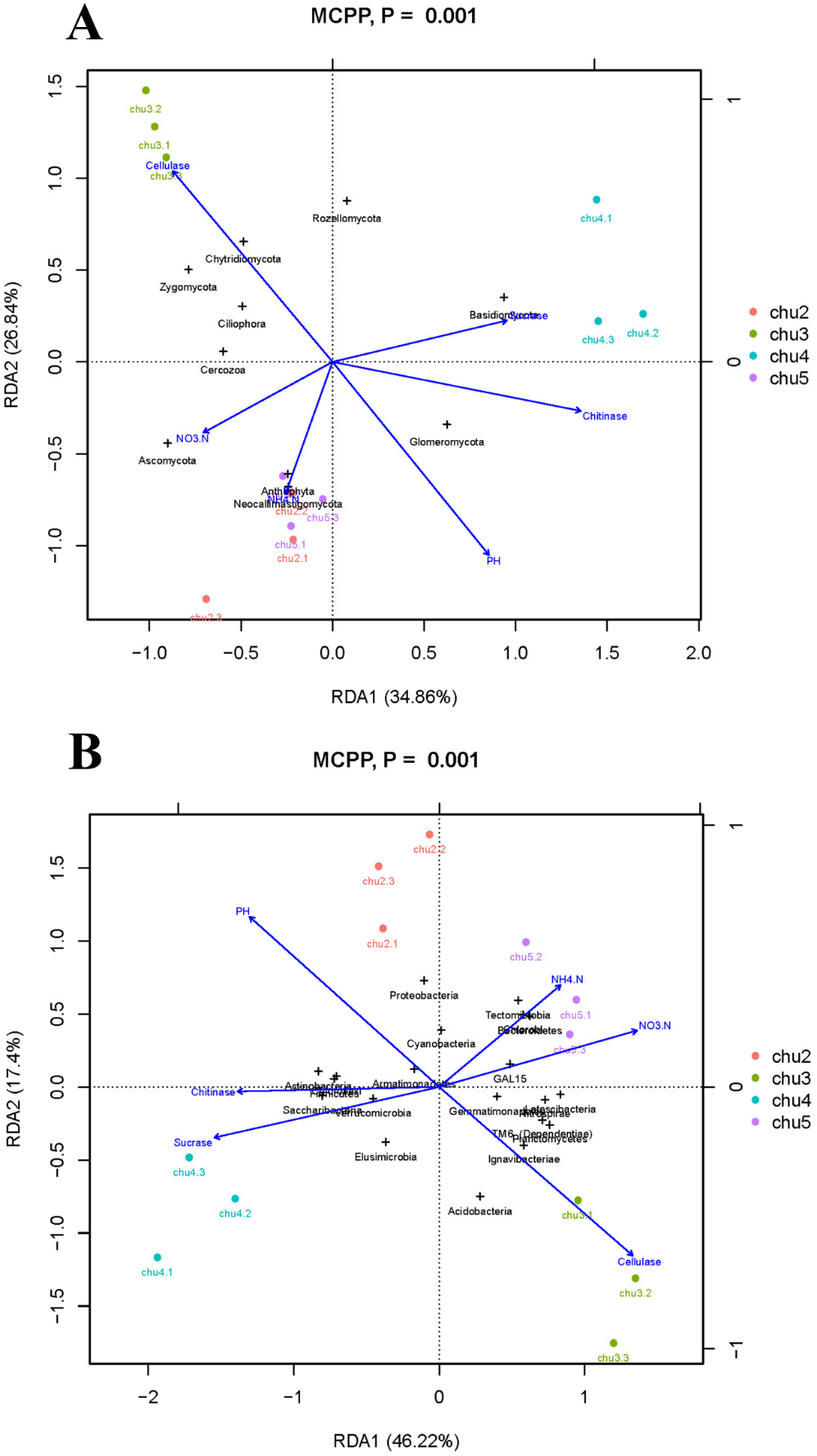
**

**Figure S9** Redundancy analysis (RDA) analysis demonstrated the relationships between soil environmental factors and microbial communities. A and B represent the correlation between the microbial taxa (fungal and bacterial phyla) and soil physiochemical properties.


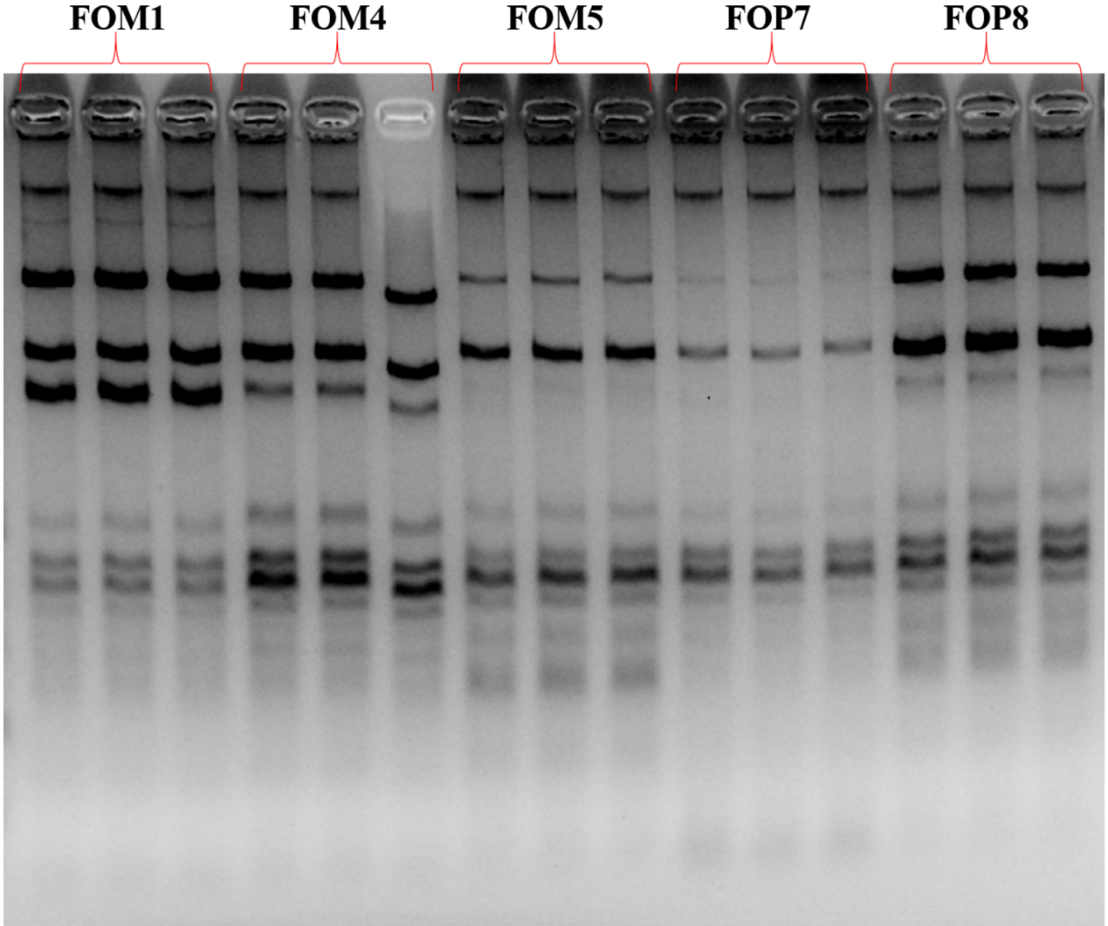


**Figure S10** SSR showed the genes amplified from 5 *F. oxysporum* strains.

**
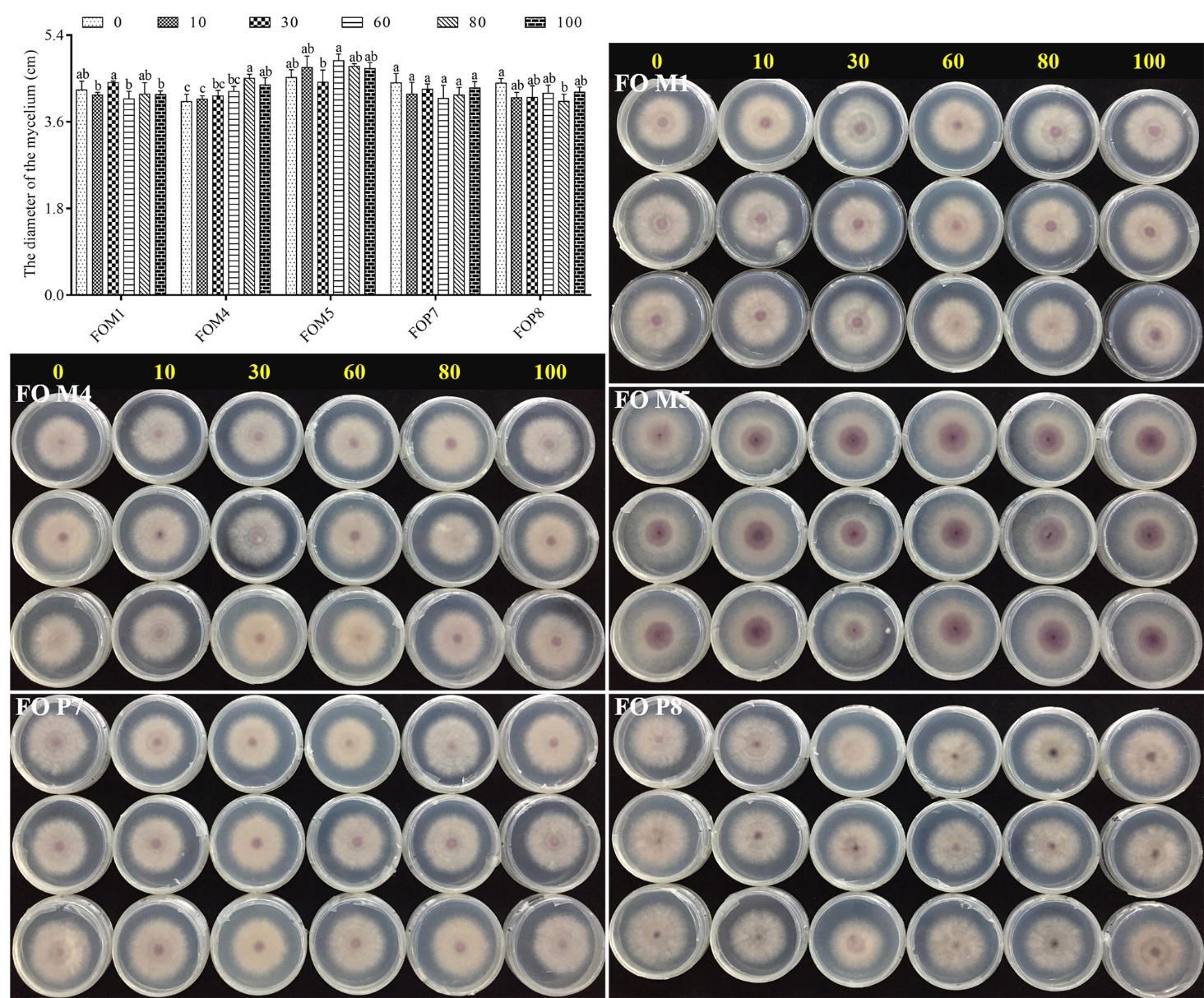
 Figure S11** The effects of gallic acid on the growth of 5 *Fusarium oxysporum* strains. The numbers represent the concentration of phenolic acids mixture (μmol/L). Columns with different letters are statistically different (LSD test, *p* < 0.05, n = 3).

**
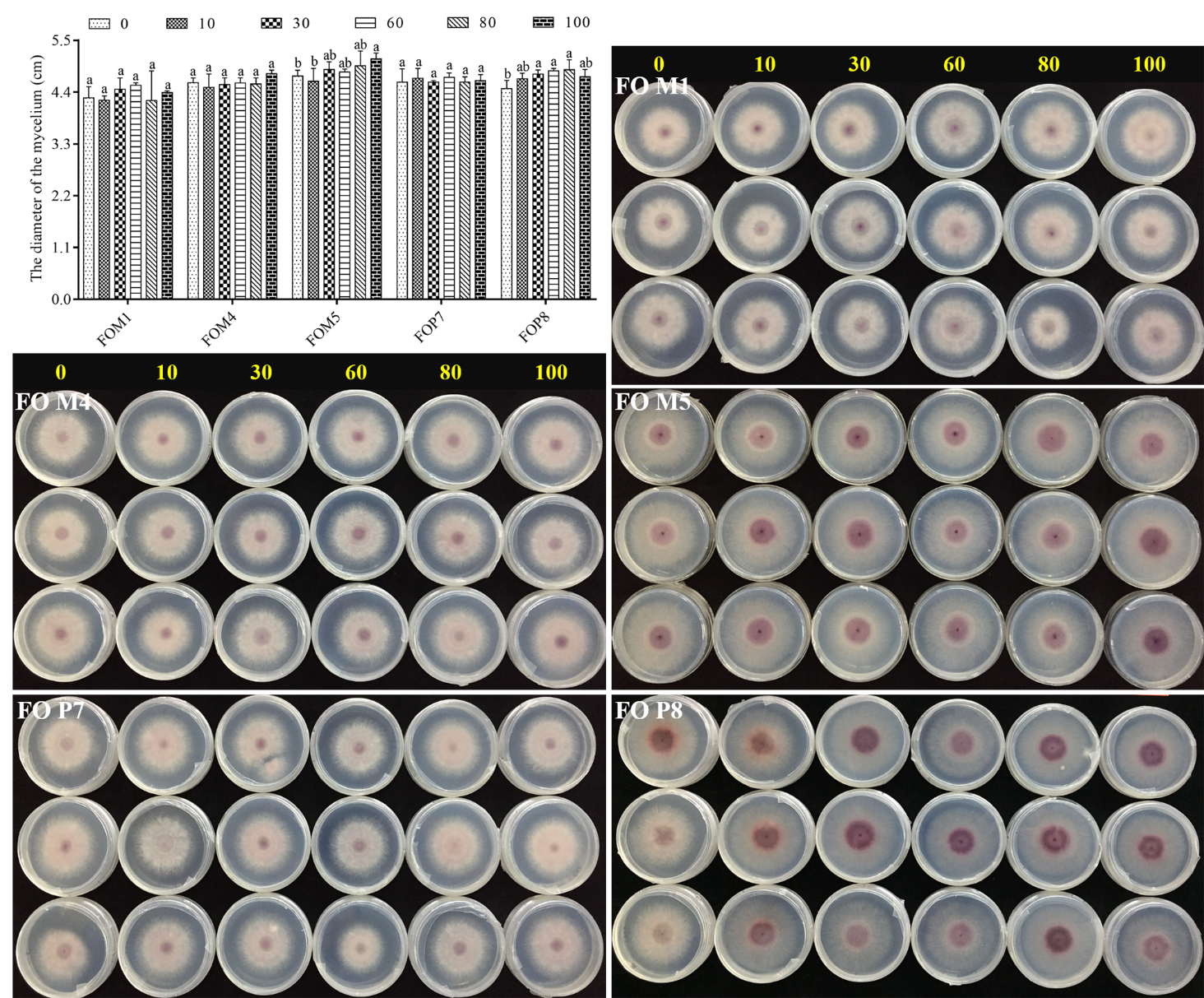
 Figure S12** The effects of the coumaric acid on the growth of 5 *Fusarium oxysporum* strains. The numbers represent the concentration of phenolic acids mixture (μmol/L). Columns with different letters are statistically different (LSD test, *p* < 0.05, n = 3).

**
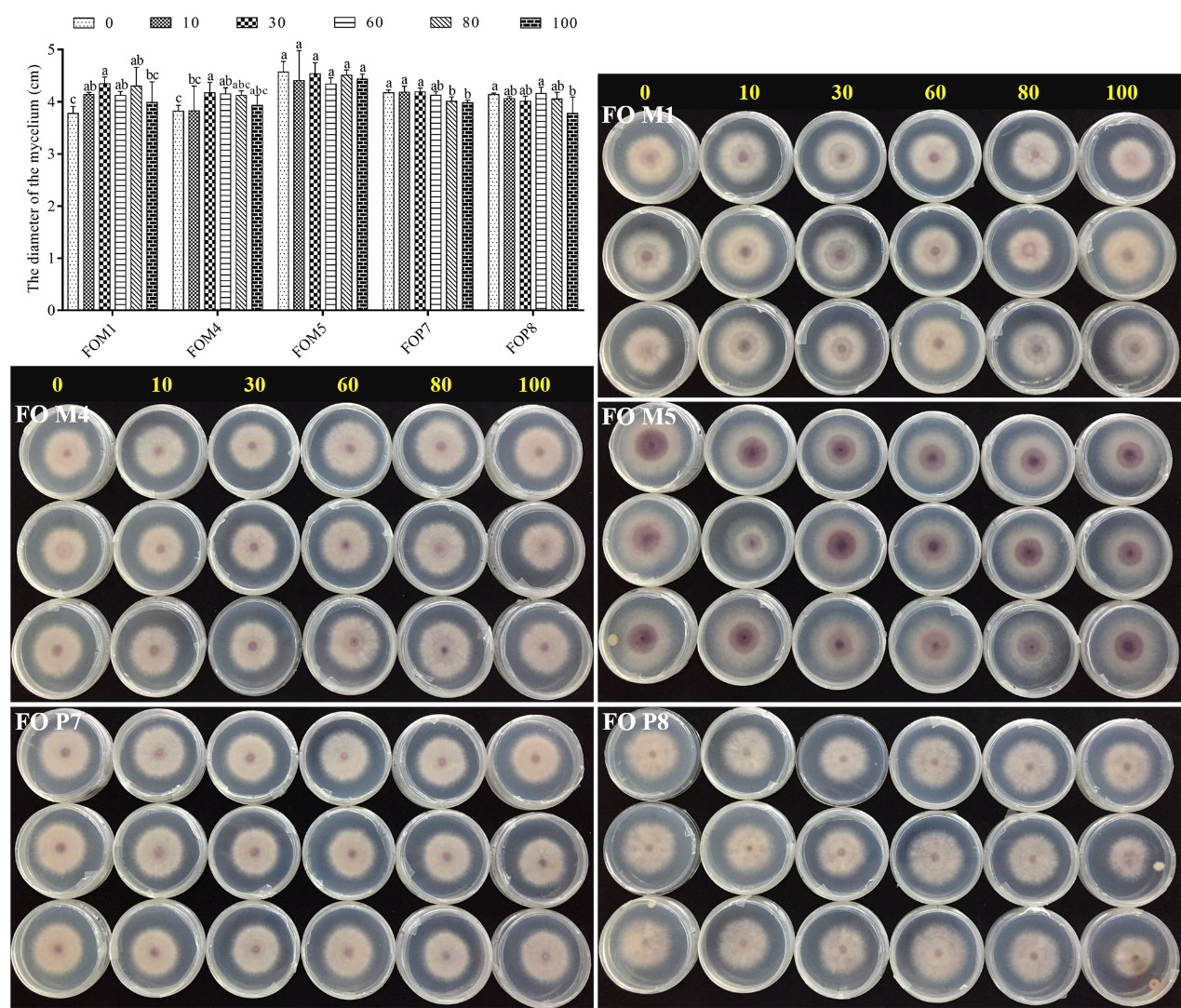
**

**Figure S13** The effects of the protocatechuic acid on the growth of 5 *Fusarium oxysporum* strains. The numbers represent the concentration of phenolic acids mixture (μmol/L). Columns with different letters are statistically different (LSD test, *p* < 0.05, n = 3).

**
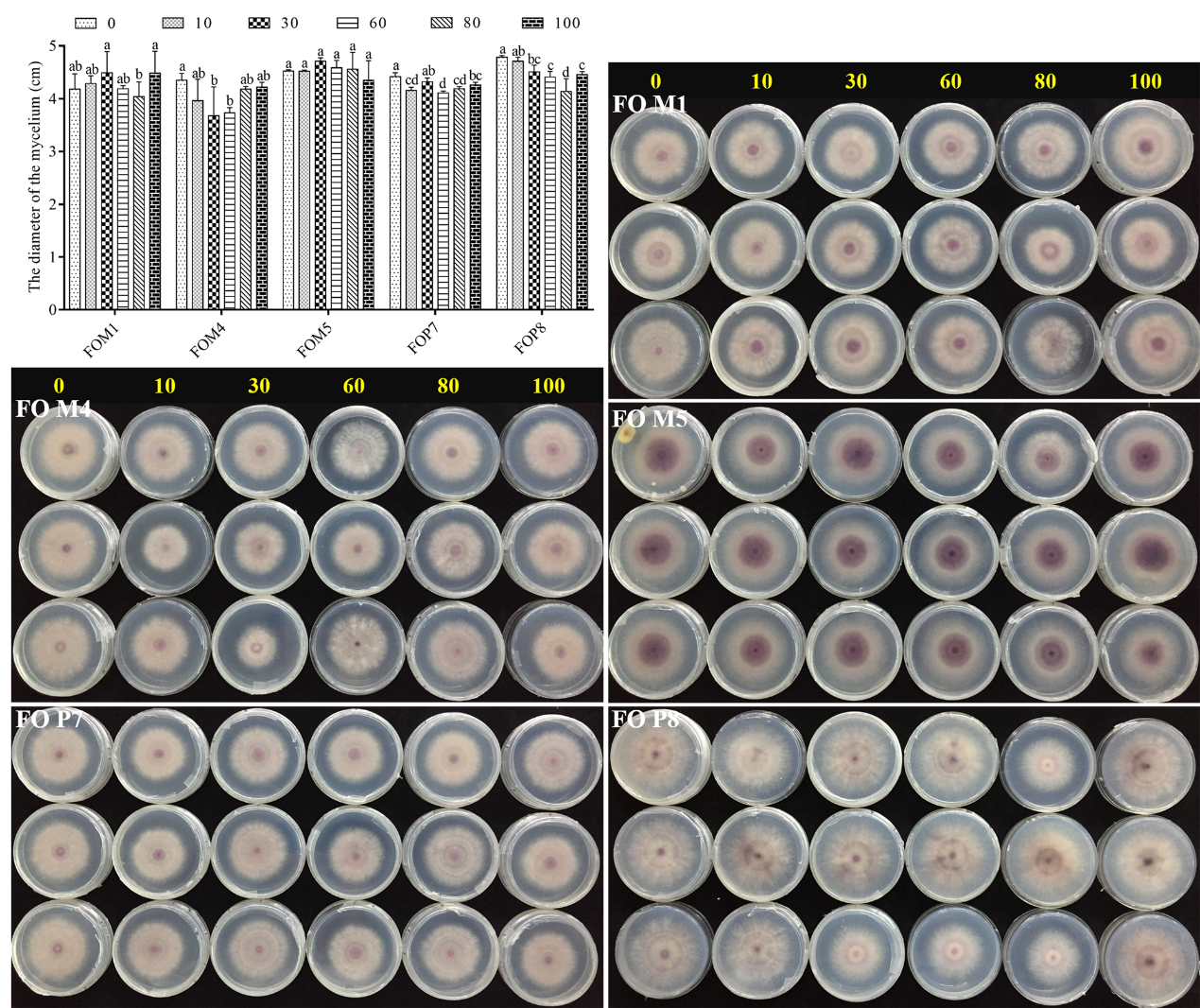
**

**Figure S14** The effects of the p-hydroxybenzoic acid on the growth of 5 *Fusarium oxysporum* strains. The numbers represent the concentration of phenolic acids mixture (μmol/L). Columns with different letters are statistically different (LSD test, *p* < 0.05, n = 3).

**
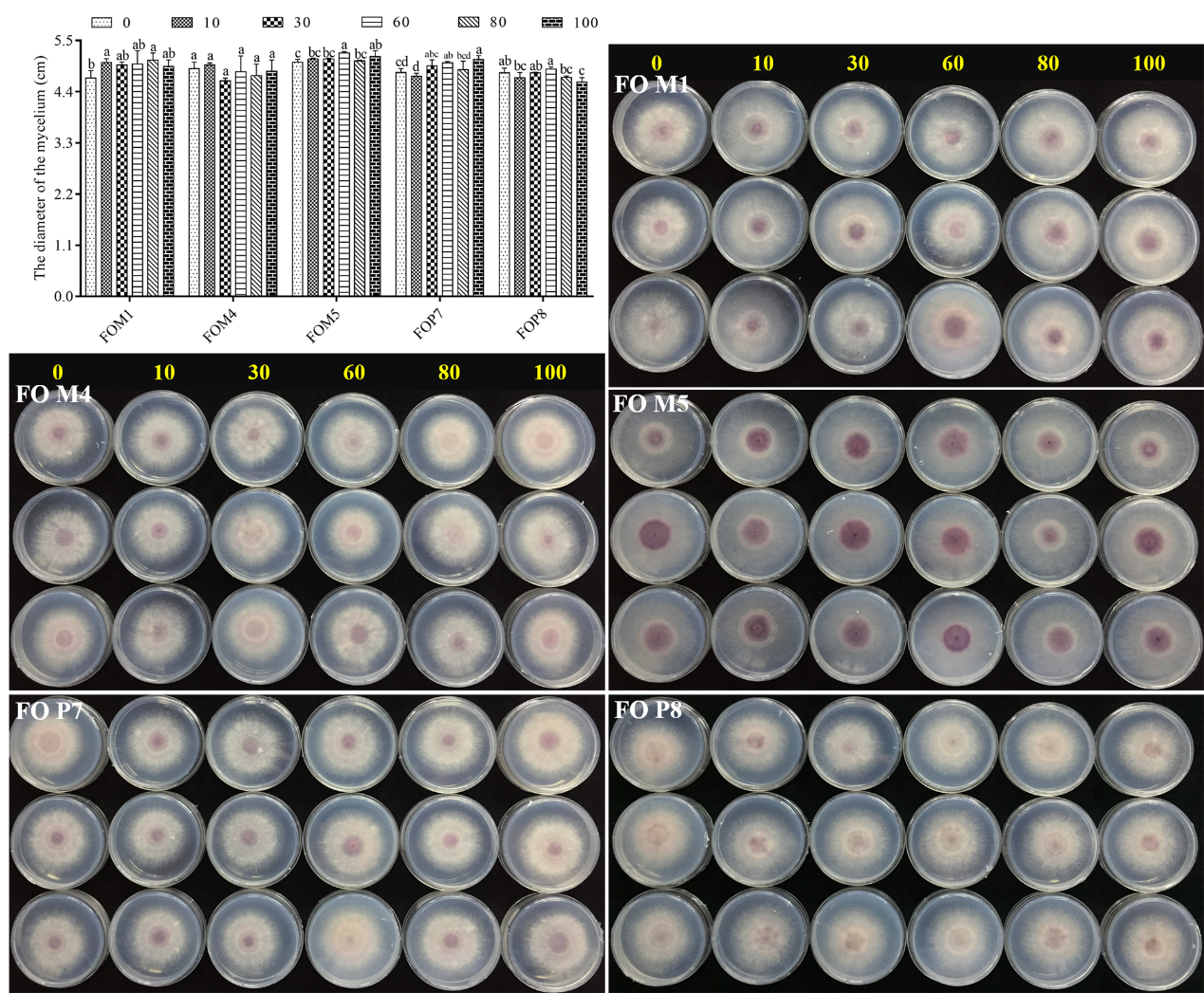
**

**Figure S15** The effects of the syringic acid on the growth of 5 *Fusarium oxysporum* strains. The numbers represent the concentration of phenolic acids mixture (μmol/L). Columns with different letters are statistically different (LSD test, *p* < 0.05, n = 3).

**
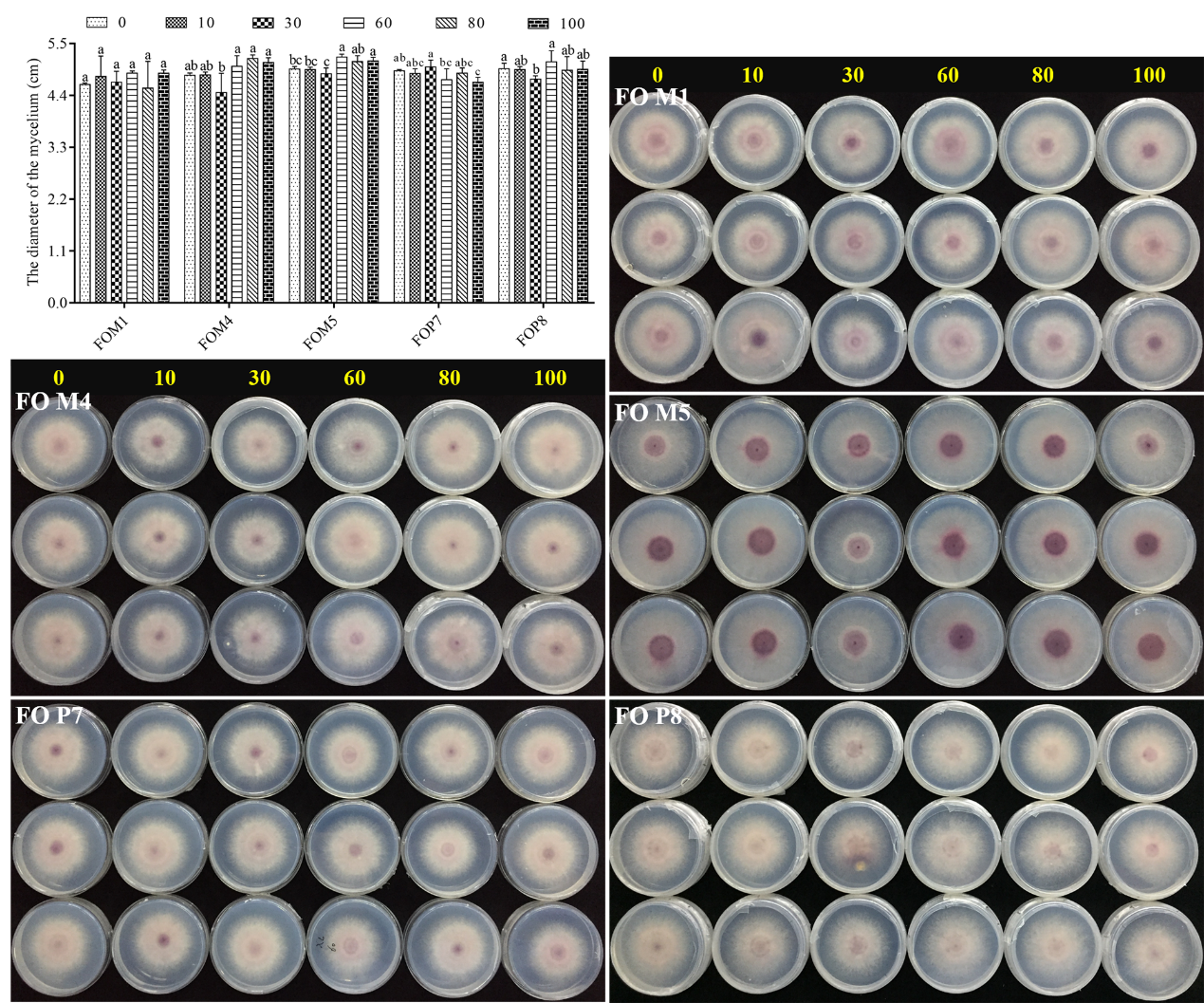
**

**Figure S16** The effects of the vanillin on the growth of 5 *Fusarium oxysporum* strains. The numbers represent the concentration of phenolic acids mixture (μmol/L). Columns with different letters are statistically different (LSD test, *p* < 0.05, n = 3).

**
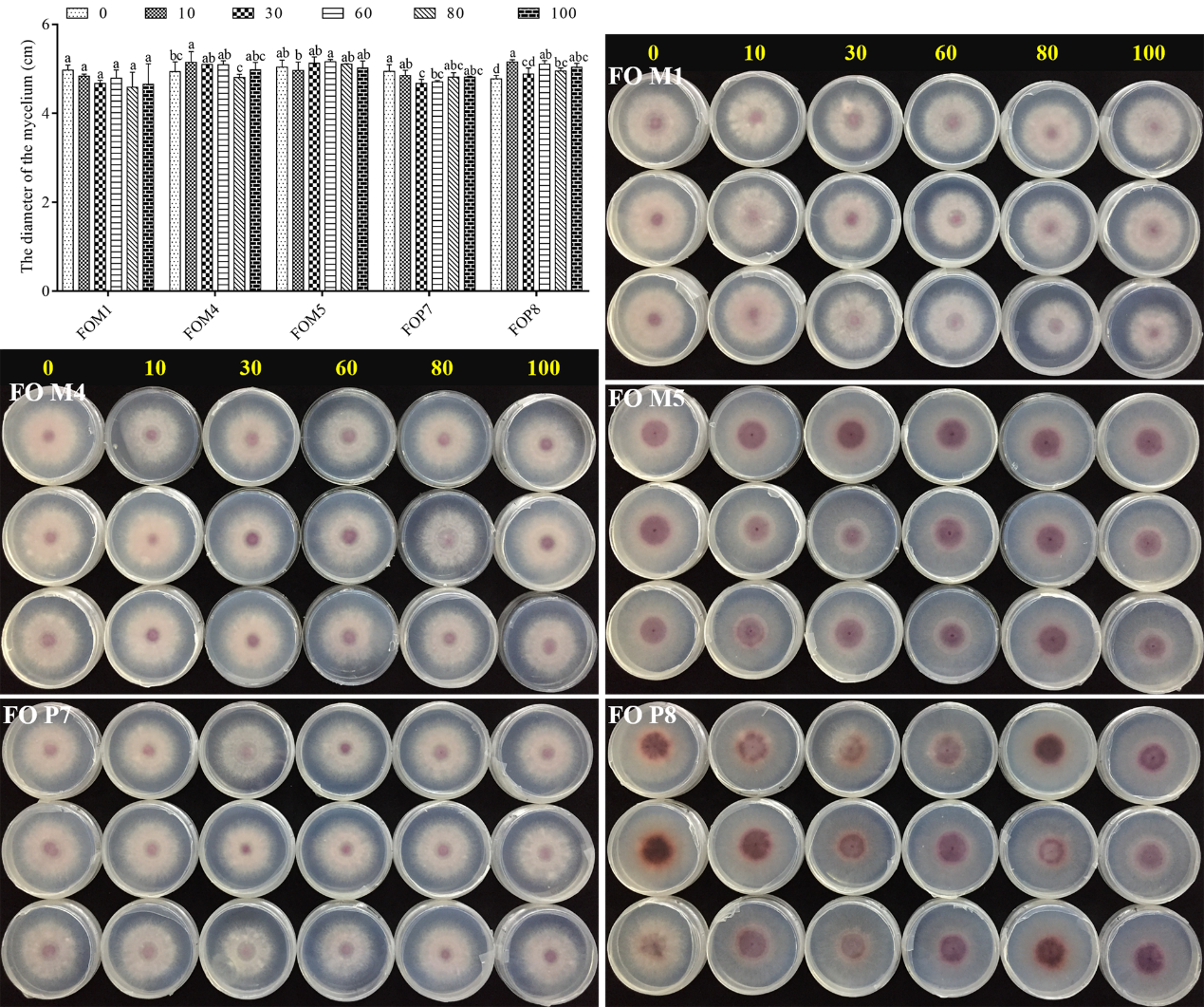
**

**Figure S17** The effects of the ferulic acid on the growth of 5 *Fusarium oxysporum* strains. The numbers represent the concentration of phenolic acids mixture (μmol/L). Columns with different letters are statistically different (LSD test, *p* < 0.05, n = 3).

**
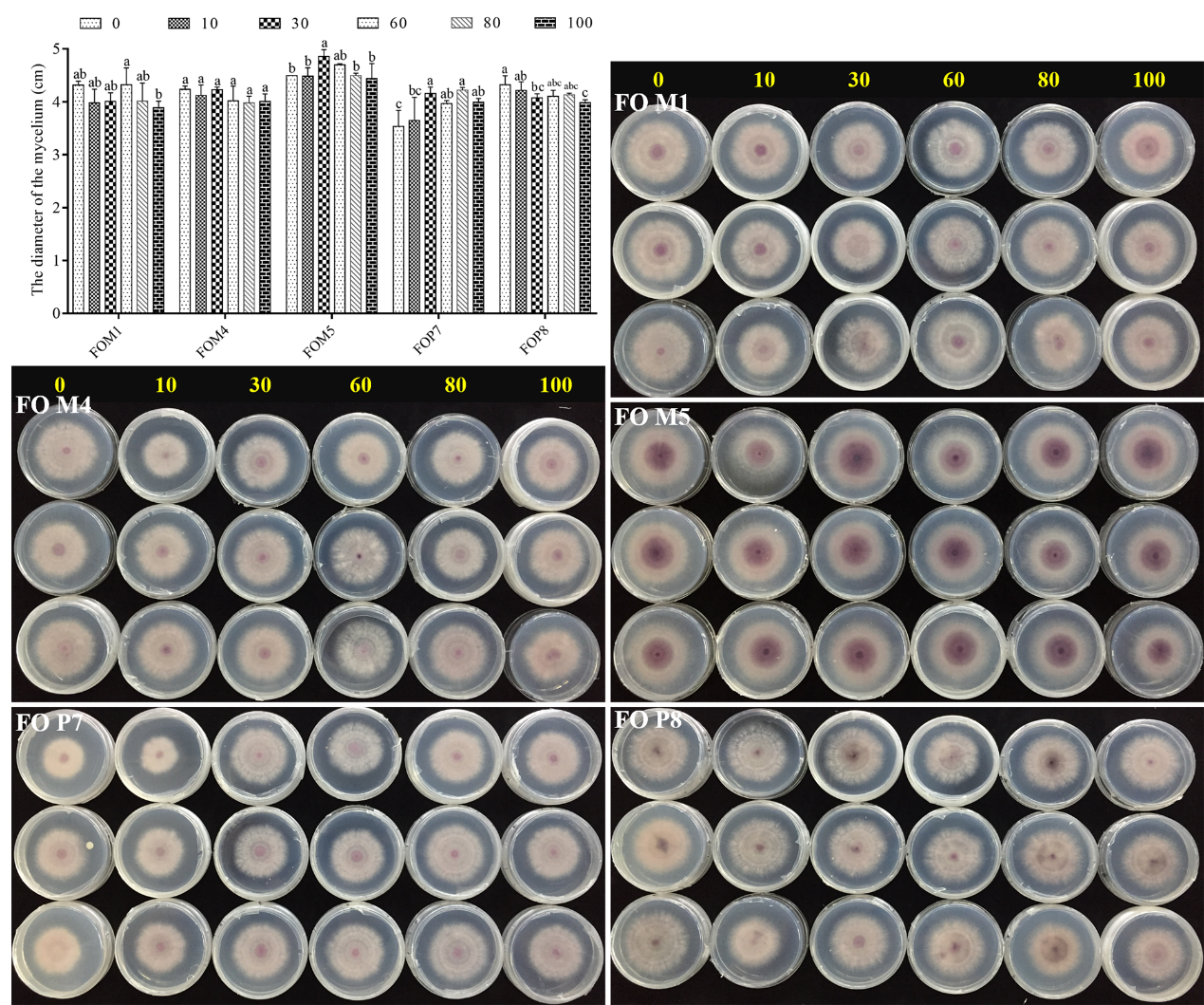
**

**Figure S18** The effects of the benzoic acid on the growth of 5 *Fusarium oxysporum* strains. The numbers represent the concentration of phenolic acids mixture (μmol/L). Columns with different letters are statistically different (LSD test, *p* < 0.05, n = 3).

**References**

Gao Z, Li B, Zheng C & Wang G (2008) Molecular detection of fungal communities in the Hawaiian marine sponges Suberites zeteki and Mycale armata. *Applied and Environmental Microbiology* **74**: 6091-6101.

Ikoyi I, Fowler A & Schmalenberger A (2018) One-time phosphate fertilizer application to grassland columns modifies the soil microbiota and limits its role in ecosystem services. *Science of The Total Environment* **630**: 849-858.

Kielak A, Pijl AS, Van Veen JA & Kowalchuk GA (2008) Differences in vegetation composition and plant species identity lead to only minor changes in soil-borne microbial communities in a former arable field. *FEMS microbiology ecology* **63**: 372-382.

Lievens B, Brouwer M, Vanachter A, Levesque CA, Cammue BPA & Thomma B (2005) Quantitative assessment of phytopathogenic fungi in various substrates using a DNA macroarray. *Environmental Microbiology* **7**: 1698-1710.

Mahfooz S, Maurya DK, Srivastava AK, Kumar S & Arora DK (2012) A comparative in silico analysis on frequency and distribution of microsatellites in coding regions of three formae speciales of Fusarium oxysporum and development of EST–SSR markers for polymorphism studies. *FEMS microbiology letters* **328**: 54-60.

Okubo A & Sugiyama S-i (2009) Comparison of molecular fingerprinting methods for analysis of soil microbial community structure. *Ecological research* **24**: 1399-1405.

Onwuchekwa NE, Zwiazek JJ, Quoreshi A & Khasa DP (2014) Growth of mycorrhizal jack pine (Pinus banksiana) and white spruce (Picea glauca) seedlings planted in oil sands reclaimed areas. *Mycorrhiza* **24**: 431-441.

T Weedon J, A Kowalchuk G, Aerts R, van Hal J, van Logtestijn R, Taş N, FM Röling W & M van Bodegom P (2012) Summer warming accelerates sub‐arctic peatland nitrogen cycling without changing enzyme pools or microbial community structure. *Global Change Biology* **18**: 138-150.

Yao Q, Liu J, Yu Z, Li Y, Jin J, Liu X & Wang G (2017) Three years of biochar amendment alters soil physiochemical properties and fungal community composition in a black soil of northeast China. *Soil Biology Biochemistry* **110**: 56-67.
